# Life-Cycle Emissions and Human Health Implications of Multi-Input, Multi-Output Biorefineries

**DOI:** 10.1021/acs.est.4c12920

**Published:** 2025-08-28

**Authors:** Sarah L. Nordahl, Melissa Moore, Nawa Raj Baral, Wilson McNeil, Yan Wang, Corinne D. Scown

**Affiliations:** † Energy Technologies Area, Lawrence Berkeley National Laboratory, 1 Cyclotron Road, Berkeley, California 94720, United States; ‡ Department of Civil and Environmental Engineering, 1438 University of California, Berkeley, Berkeley, California 94720, United States; § Joint BioEnergy Institute, 5885 Hollis Street, Emeryville, California 94608, United States; ∥ Biosciences Area, 1666 Lawrence Berkeley National Laboratory, 1 Cyclotron Road, Berkeley, California 94720, United States; ⊥ Energy & Biosciences Institute, University of California, Berkeley, Berkeley, California 94720, United States

**Keywords:** anaerobic digestion, biofuels, bioproducts, waste management, air pollution, eutrophication, acidification

## Abstract

To meaningfully broaden the supply of fuels for the transportation sector, biofuel production must be scaled up and this requires a wider array of biomass feedstocks, including agricultural residues and organic waste. Rather than pursuing conversion of lignocellulosic biomass to fuels and anaerobic digestion of wastes as separate pathways, there are economic and environmental advantages associated with integrating these processes in a single facility. However, existing research rarely goes beyond carbon footprints in quantifying the effects of such a shift in bioenergy production. In addition to CO_2_, CH_4_, and N_2_O, this study explores the life-cycle air pollution (NH_3_, volatile organic compounds, NO_*x*_, SO_2_, and PM_2.5_), marine eutrophication, acidification, and local external cost implications of biorefineries capable of taking in crop residues, food waste, and manure to produce liquid fuel, electricity, and/or other options such as renewable natural gas (RNG), hydrogen, bioplastics, and protein-rich livestock feed. Relative to a single-input, single-output baseline, biorefineries integrated with organic waste codigestion to coproduce electricity or RNG can reduce life-cycle CO_2_-equivalent emissions by 84–149%, and the monetized external impacts across all scenarios range from $1.07/gallon to −$0.75/gallon ethanol.

## Graphical abstract

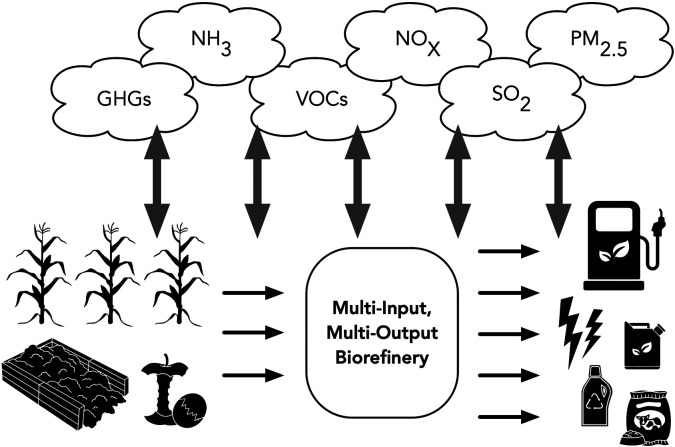

## Introduction

Energy-dense renewable fuels are essential to achieving emissions reductions across the global economy.^1^ In the U.S., the most widely used biofuel feedstocks, starches and lipids, do not currently provide the deep emissions reductions and adequate supply needed to meet the transportation sector’s needs. To do so, lignocellulosic feedstocks will need to be converted to liquid fuels.^2^ However, a variety of technical and economic challenges, as well as incentive design choices, have so far resulted in negligible volumes of lignocellulosic fuel production.^3,4^ Nearly all of the cellulosic biofuel volume as defined in the United States (U.S.) Environmental Protection Agency’s Renewable Fuel Standard (RFS) is renewable natural gas (RNG) originating from upgraded biogas and intended for use in compressed natural gas (CNG) vehicles.^5^ Rather than continuing to pursue anaerobic digestion (AD) as a separate pathway to a transportation fuel (i.e., RNG), there are several advantages associated with integrating lignocellulosic biorefineries with AD in a single facility.^6^ This study explores the life-cycle air pollution, greenhouse gas (GHG), and external cost implications of re-envisioned biorefineries capable of taking in crop residues, food waste, and manure to produce liquid fuel and electricity or other options such as RNG, hydrogen, bioplastics, and protein-rich livestock feed. Such a strategy would leverage the onsite wastewater treatment at biorefineries and increase the diversion of wastes that are known to drive anthropogenic CH_4_ and air pollutant emissions, while diversifying both the inputs and outputs of future biorefineries through the integration of AD.

While AD is not strictly required for biomass conversion to products like bioethanol, it is useful for managing residual material left after conversion and improving facility economics, particularly for facilities with wastewater that contains solvents and other chemicals that make the recovered organics and nutrients unsuitable for animal feed.^7^ AD is standard practice in wastewater treatment facilities, and large-scale lignocellulosic biorefineries may opt to build these as part of their onsite wastewater treatment and recycling.^8^ A review from Jarunglumlert and Prommauk demonstrates how AD and the coproduction of biogas alongside bioethanol at lignocellulosic biorefineries can reduce the minimum selling price of the finished fuel.^6^ Most commonly, biogas can serve as a fuel for onsite generation of heat and/or electricity.^6,7^ Excess biogas can be upgraded to RNG and injected into pipelines for sale via book-and-claim or sold to utilities that must meet biomethane procurement targets, as is the case for investor-owned utilities in California.^9^

Although AD may be used solely to treat biorefinery wastewater, digesters can often accommodate additional solids. It is possible to codigest locally available organic waste streams that would otherwise be landfilled or discharged to land and water in a manner that contributes to eutrophication. The digestibility and nutrient availability vary across feedstocks, and diversifying feedstocks can improve biogas yields.^10–13^ Viable secondary inputs for a codigestion system include organic municipal solid waste, food processor waste, manure, and wastewater treatment sludge.^7^ For instance, Aboudi et al. found that codigesting sugar beet residues with swine or cattle manure increased CH_4_ production by 70 and 31%, respectively.^14^ Anjum et al. found that codigesting corn stover with food waste more than doubled biogas yields relative to digesting food waste alone.^15^

With enhanced biogas yields, biorefineries can satisfy onsite energy demand and use surplus biogas to produce additional saleable outputs. Excess biogas from codigestion can be used for electricity generation, upgraded to RNG, or converted into other valuable products like bioplastics or protein for animal feed.^16^ By leveraging codigestion to generate co-products, biorefineries can further improve economic performance while delivering waste diversion benefits. Herrera Adarme et al. found that codigestion of vinasse and hemicellulose hydrolysates at a sugarcane biorefinery for energy production improves revenue while lowering carbon emissions.^17^ Wang et al. found that integrating corn stover-to-bioethanol conversion with manure and food waste codigestion to coproduce RNG or bioplastics can reduce costs and reduce life-cycle GHG emissions because of the waste diversion benefits.^16^

Despite continued interest in enabling biofuel production and research on associated GHG implications, limited research has been done on other environmental impacts from these systems, such as air quality impacts and associated human health damages. This paper builds upon work previously conducted by Wang et al. to provide such an evaluation by examining the life-cycle NH_3_, volatile organic compounds (VOCs), NO_*x*_, SO_2_, and PM_2.5_ emissions in addition to GHGs (i.e., CO_2_, CH_4_, and N_2_O) from lignocellulosic biorefineries.^16^ Beyond assessing life-cycle emissions of specific pollutants, we quantify associated impacts on eutrophication, acidification, and human health. To these ends, we provide a rigorous cradle-to-grave life-cycle assessment (LCA) of five biorefinery configurations: one single-product corn stover-to-bioethanol facility and four multi-output configurations involving codigestion of manure and food waste. The multi-output configurations vary based on biogas utilization and final co-product: electricity, RNG, single-cell protein (SCP), and poly­(3-hydroxybutyrate) (PHB). In the case of biogas upgrading to RNG, we consider two RNG utilization scenarios: onsite fleet fueling to offset diesel fuel use and steam methane reforming (SMR) for hydrogen production to offset natural gas use.

## Methods

The system boundary for analysis begins with the sourcing of corn stover and ends with the production of bioethanol at a biorefinery (Figure 1). We consider commercial-scale biorefinery designs sized to process 2000 dry tonnes of corn stover per day as described by Wang et al.^16^ For the single-product baseline biorefinery, corn stover is the only feedstock considered, and bioethanol is the only output. For codigestion scenarios, feedstocks include corn stover, manure, and food waste, while the resulting co-products vary by scenario. We account for the net impacts of organic waste diversion, induced inorganic fertilizer demand, and offset credits for co-products via system expansion. Our analysis is not tied to a specific facility or site, but when applicable, we use assumptions relevant to the corn belt region of the United States, where corn stover is widely available. For instance, we consider the Midwest Reliability Organization (MRO) region of the U.S. electricity grid to evaluate direct electricity consumption at the biorefinery and feedstock-supplying farms^18^ but assume an average U.S. grid mix for other upstream electricity consumption.

**Figure 1:**
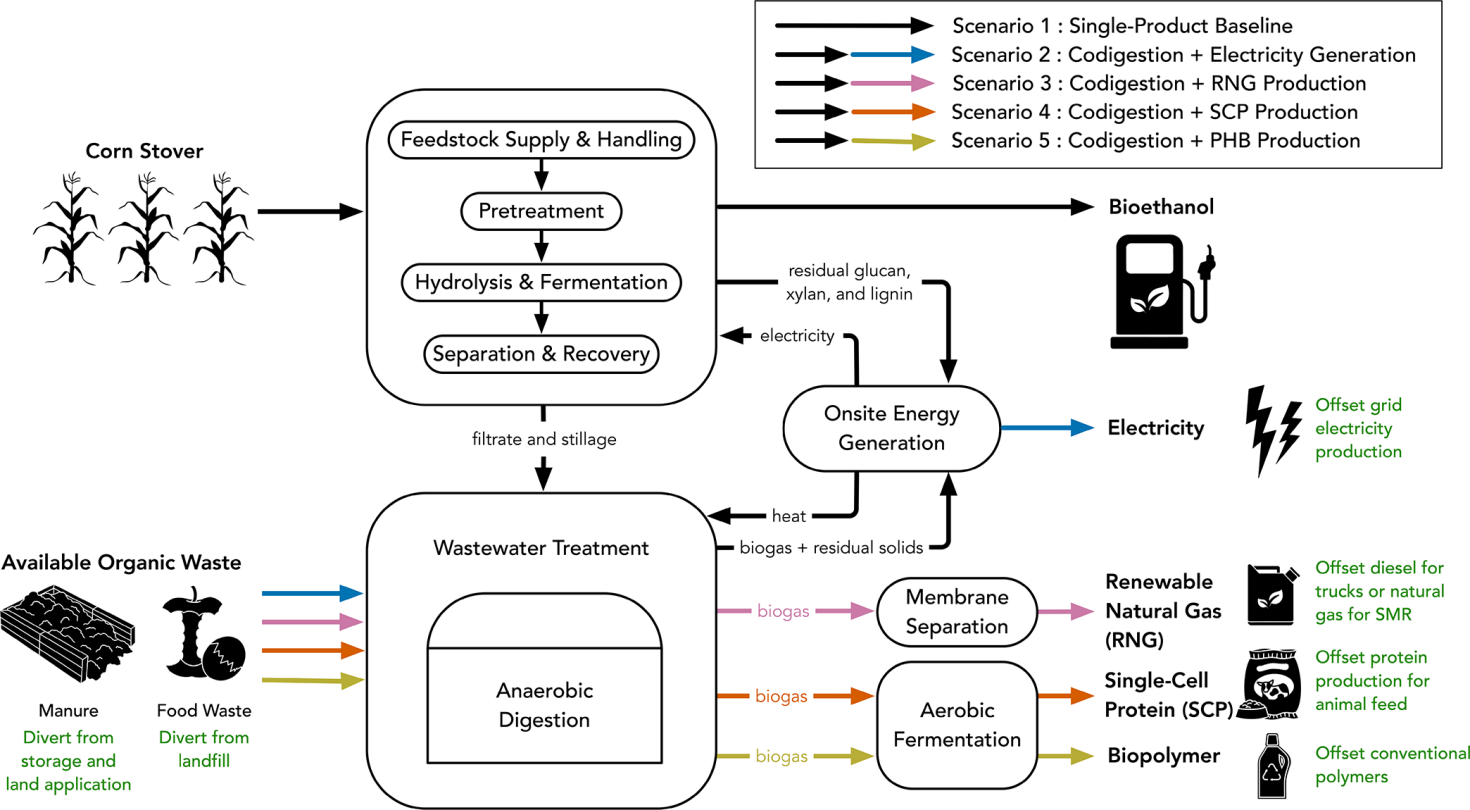
System boundary for life-cycle assessment. All scenarios include flows along the black arrows. Scenarios 2–5 include various additional flows indicated by colored arrows.

### Corn Stover-to-Ethanol Biorefinery

Corn stover and other agricultural feedstocks are converted to valuable bioenergy products, such as ethanol, via pretreatment, enzymatic hydrolysis, and fermentation at a lignocellulosic biorefinery. For pretreatment, we assume a deacetylation and mechanical refining (DMR) process.^16^ A typical facility design for such a biorefinery incorporates a wastewater treatment system equipped with AD for onsite energy generation. The wastewater treatment system includes a digester that converts the residues from corn stover processing (e.g., lignin and unconverted cellulose in filtrate) to biogas before sending residual solids for combustion. We assume a biogas leakage rate of 3.1%, the average leakage rate for a manure biodigester across all four seasons according to Flesch et al.^19^ Leaked biogas contains CH_4_, NH_3_, and H_2_S. The wastewater treatment component of the facility also directly emits flue gas containing SO_2_ to the atmosphere. CH_4_, NH_3_, H_2_S, and SO_2_ generation data are reported in Table S2 of the Supporting Information.

In our single-input facility scenario (Scenario 1), all biogas is used for electricity generation. Remaining biomass after digestion can be composted, applied to land, or combusted for additional energy recovery.^7^ We assume that remaining solids, alongside the biogas, are combusted in a boiler system to produce steam and electricity. In Scenario 1, all electricity produced onsite is consumed, and additional electricity from the grid is required. Ethanol is the only saleable output. Baseline operations can be divided into six main stages: (1) feedstock handling, (2) pretreatment, (3) enzymatic hydrolysis and fermentation, (4) ethanol recovery and separation, (5) wastewater treatment (WWT), and (6) onsite energy generation (Figure 1). Stage-specific modeling assumptions are summarized in Table S3 of the Supporting Information. Process data are adapted from Wang et al. (Figure S1, Tables S1, and S2 in the Supporting Information).^16^

### Codigestion and Organic Waste Diversion

In addition to a single-product biorefinery, we also consider multiproduct facility configurations that incorporate codigestion of organic waste to enhance biogas yields. A biorefinery with codigestion will rely on the local availability of organic waste feedstocks. In the corn belt region, swine and dairy manure are the most readily available organic waste feedstocks.^20,21^ Food waste is also available, but in lower abundance since population density and municipal waste collection are limited in rural areas.^22^ For this analysis, we assume that swine manure, dairy cattle manure, and food waste are digested alongside residual corn stover material in the WWT system of the facility. We assume that beef cattle manure and poultry manure, which are not commonly processed in anaerobic digesters today, are not available as feedstocks. We use organic waste loading rates and transportation assumptions from Wang et al. and the U.S. Billion-Ton Report (see Table S1 in Supporting Information).^16,23^

There are substantial benefits associated with diverting organic waste from conventional end-of-life pathways. We assume that food waste is diverted from landfills, where it would have anaerobically decomposed, contributing to CH_4_ emissions (even in landfills with gas capture systems). Landfilling emission factors used for assessing avoided emissions from food waste are reported in Table S5 of the Supporting Information. Manure diversion avoids long-term manure storage and land application, both of which are associated with GHG and air pollution emissions.^24,25^ We assume that all diverted manure would otherwise be stored outdoors or in unenclosed areas where emissions and odors are vented directly to the atmosphere, as is common practice in the U.S. We use CH_4_, N_2_O, and NH_3_ emission factors that are specific to swine and dairy cattle to estimate avoided emissions resulting from the diversion of manure from a weighted-average range of baseline practices to an anaerobic digester.^26–28^ Manure storage systems can vary operation-to-operation and can involve slurry tanks, deep pits, and open lagoons.^29^ Many studies treat these storage systems as mutually exclusive options, but farms commonly employ a combination of these storage processes before field application.^30,31^ Wang et al. and Owen and Silver review emission measurements from a variety of technologies and management scenarios, demonstrating that manure management emissions can be highly variable.^27,32^ From the data provided in these two reviews, we identify conservative emission factors for managing untreated liquid manure to avoid overestimating the benefits of manure diversion. For dairy manure, we use CH_4_, N_2_O, and NH_3_ emission factors from Amon et al. based on dairy manure during slurry tank storage and field application to grassland.^26^ For swine manure, we use CH_4_, N_2_O, and NH_3_ emission factors for deep pit storage and application to paddy fields from Wang et al.^27^ We assume that CO_2_ emissions from manure are biogenic and therefore are not included in the total 100-year global warming potential. To estimate avoided VOC impacts, we use an average VOC emission factor for outdoor manure “composting” (dry storage) from Nordahl et al. as a reasonable proxy for VOC emissions across all stages of manure management (including scraping/storage from wet storage) and apply it to both dairy cattle and swine manure.^33^ Because of a lack of data, we do not consider NO_*x*_, SO_2_, and PM_2.5_ emissions from manure storage or application. These pollutants are typically associated with combustion and are not expected to be substantial contributors to air pollution impacts from baseline manure management practices. All emission factors are reported in Table S5 of the Supporting Information. To compensate for the diversion of manure from use as a soil amendment, we consider the induced demand for inorganic fertilizer. We assume that urea will be used as a 1:1 replacement for manure based on nitrogen content. We assume nitrogen contents (g N/kg manure) of 6.19 and 5.23 for swine manure and dairy cattle manure, respectively.^34^

### Biogas Utilization and Offset Credits

For the codigestion scenarios, we model four possible biogas utilization pathways and associated co-products: electricity, RNG, PHB, and SCP. Some of these co-products may displace a variety of different products in real-world markets. PHB is a compostable plastic, and SCP can serve as aquaculture feed. The specific products that they could displace are uncertain. We selected conservative options when assigning displacement credits for these co-products. Facility designs and related process data for these scenarios in addition to Scenario 1 are taken from Wang et al.^16^

In the case of electricity production without other co-product generation (Scenario 2), the facility configuration is the same as that in Scenario 1: there is an onsite energy generation component of the system that is equipped with CHP units. While all electricity generated by the CHPs is consumed onsite in Scenario 1, Scenario 2 produces enough electricity from enhanced biogas production to satisfy onsite energy needs and export surplus (about 0.03 kWh per MJ ethanol) to the grid.^16^ We assume that surplus electricity offsets emissions from the MRO grid region, which spans the corn belt. Exports would be consistent on an hourly basis, effectively operating as the baseload power. Because untreated biogas cannot be stored in a compressed form and industrial CHP units are designed to operate consistently, we assume that the facility does not engage in load-following or the provision of any ancillary services.

In Scenario 3, biogas is cleaned, upgraded, and compressed to an RNG. We assume membrane separation as the biogas upgrading method (Figure 1). There are multiple applications for RNG.^16,35^ For this study, we model two RNG utilization scenarios: truck fleet fueling in place of diesel (Scenario 3A) and SMR to produce hydrogen offsetting natural gas (Scenario 3B). For Scenario 3A, we assume that 1.3 L of diesel is equivalent to 1 kg of RNG based on energy content. To account for differences in combustion emissions between RNG and diesel, we use fuel-specific emission factors for heavy-duty trucking, assuming that CO_2_ emissions from RNG are biogenic (see Table S5 in the Supporting Information). For Scenario 3B, we assume that RNG is used in place of natural gas for a hydrogen production process via SMR. We assume that RNG replaces natural gas on a 1:1 basis. Additionally, CO_2_ emissions from SMR using RNG are biogenic versus fossil-based CO_2_ emissions from natural gas use.^36^ We assume that using RNG avoids 2.9 kg of direct CO_2_ emissions per kg of fuel input to SMR compared to using natural gas without a carbon capture system.^36^

Scenarios 4 and 5 both involve microbial conversion and aerobic fermentation of biogas to produce bioproducts. The process stages include cell growth, PHB accumulation, dewatering, mechanical disruption, centrifugation, and separation. The final product is either SCP, as in Scenario 4, or PHB, as in Scenario 5. The primary difference in the process between producing SCP and PHB is the removal of methanotrophic cells in the cell growth and PHB accumulation stages for SCP production. SCP can be used as a protein-rich livestock feed.^37,38^ SCP may offset demand for fishmeal protein, which is similar in composition and emissions-intensive to produce, or other common sources of feed protein like soybean meal.^39,40^ Because actual displacement induced by SCP availability in a realistic market is unknown, we credit SCP with offsetting soybean meal, which is more widely available than fishmeal, on a 1:1 basis with respect to the protein content. This is consistent with other LCAs looking at similar SCP-producing systems.^39,41^ We assume protein contents of 72 and 44% for SCP and soybean meal, respectively.^16,42–44^

PHB is a compostable bioplastic that is primarily used for packaging. Because realistic material substitution factors between fossil-based plastics and bioplastics are unknown, we consider two possible displacement scenarios: Scenario 5A, in which PHB offsets polylactic acid (PLA), the most widely produced compostable bioplastic today, and Scenario 5B, in which PHB offsets polypropylene (PP), a fossil-based plastic with comparable properties and applications.^16,45^ In this cradle-to-gate analysis, we assume that PHB offsets either PLA or PP on a 1:1 ratio based on mass because the materials have similar melting temperatures, PHB is commonly blended with either PLA or PP to improve material performance, and there is likely available market capacity for blending with PHB.^46–50^ Although we do not explicitly incorporate end-of-life management and emissions in this analysis, the differences in end-of-life emissions between PHB waste and PLA or PP waste are likely to be negligible. Landfilling remains the dominant end-of-life pathway for plastics, as most plastic packaging is screened out in composting operations, and both PHB and PLA can reasonably be expected to degrade far more slowly than other organics, such as food waste, thus emitting minimal CH_4_ in landfills. PP, which is not designed for biodegradability, degrades even more slowly than bioplastics in landfills.^51^ We use life-cycle emission factors from Vink et al.^52^ for PLA production and life-cycle inventory data from Franklin Associates (2011)^53^ to assess PP production. It is important to note that assumptions regarding co-product offset credits can have a substantial impact on the results, but are widely uncertain and subject to dynamic market behavior.

### Life-Cycle Assessment

The functional unit for the cradle-to-gate LCA is 1 MJ of bioethanol produced. We use a physical unit-based input–output LCA model, Agile-Cradle-to-Grave (Agile-C2G),^54^ to assess GHG, NH_3_, VOC, NO_*x*_, SO_2_, and PM_2.5_ footprints for five main biorefinery configurations (Scenarios 1–5). This model relies on a large input–output table that includes all relevant processes, products, and services with associated physical units (e.g., kWh for electricity) and a series of impact vectors for each type of emission based on the direct emissions per physical unit output for that process/product/service. All relevant inventory data has been included in the Supporting Information. For GHG footprints, we exclude biogenic CO_2_ and consider nonbiogenic CO_2_, CH_4_, and N_2_O. In the multi-output scenarios, we employ system expansion to credit co-products for displacing conventional products and avoiding associated emissions. Primary life-cycle inventory and process data primarily comes from Wang et al.^16^ The LCA model is populated with the best available emission factors and input–output data from the literature (Tables S5 and S6 in the Supporting Information). For the GHG analysis, we also forecast future impacts based on projected changes in grid electricity production. Forecasts for future carbon intensities of electricity are based on two of the U.S. electricity sector scenarios from the National Renewable Energy Laboratory’s (NREL’s) Cambium data: one standard “mid-case” with average costs and one “high renewables” case with low costs for renewable energy and batteries.^55^ For forecasting, unlike the current-day analysis, we treat direct and upstream electricity consumption the same and consider the average U.S. electricity grid mix to evaluate the associated GHG impacts. In addition to modeling life-cycle emissions of specific pollutants, we evaluate the life-cycle impacts of each scenario on marine eutrophication and acidification potential using characterization factors from the Tool for Reduction and Assessment of Chemicals and Other Environmental Impacts (TRACI).^56,57^ See Section 3 in the Supporting Information for detailed information.

Because there are uncertainties and variability associated with several components of our analysis, we also conduct a sensitivity analysis of our LCA results using Monte Carlo simulations. We create probability distributions for key emission factors (Tables S9 and S10), which are sampled from during 5,000 Monte Carlo simulations of our LCA model. As discussed further in the results, the manure management counterfactual (and associated emissions) is a key driver of uncertainty across GHG emissions, air pollution and human health, and eutrophication impacts. Uncertainty regarding the use of co-products and associated benefits is not captured in our sensitivity analysis. Instead, we capture a range of possible life-cycle impacts from integrated biorefineries with a rigorous scenario analysis. See Section 4 in the Supporting Information for additional details.

### Local Human Health Damages and External Costs

To understand the trade-offs between GHG and local air pollution impacts, we convert applicable gaseous emission results from the life-cycle assessment into monetary values. We assume a monetary value of carbon equal to $185 (in 2020 USD) per tonne of CO_2eq_.^58^ For non-GHG emissions, we use the InMAP Source-Receptor Matrix (ISRM) to estimate external costs by considering human health impacts from air pollution exposure, which are converted to a dollar value based on the value of statistical life (VSL) assumed to be 9 million USD.^59^ The ISRM models changes in PM concentrations and associated mortality cost by considering precursor emissions (i.e., NH_3_, VOC, NO*_X_*, and SO_2_) and secondary PM_2.5_ formation. We do not include eutrophication or acidification impacts in this cost analysis because there are no reasonable estimates of cost factors to appropriately convert these environmental impacts into monetary damages in USD.

Human health damages from air pollution are location-specific because they depend on background atmospheric concentrations, meteorological conditions, and local population density. This study does not include the human health damages associated with upstream impacts or co-product offsets because the specific locations of those emissions are difficult to assign and highly case-specific (e.g., natural gas extraction sites and petroleum refineries). Instead, to understand the local external costs, we consider only direct impacts from the life-cycle that we can appropriately assume occur in the facility region. The following components of the life-cycle are included: direct emissions from corn stover collection, feedstock transportation, direct facility emissions, manure diversion, and landfill diversion. We estimate damages for two example biorefinery locations: Iowa (a realistic, possible location for a corn stover biorefinery) and California (to depict how damages may differ in another region). For both cases, we used JBEI’s BioSiting webtool to identify the regions in the state that are rich in manure and agricultural resources for the biorefinery location.^21^ We assume that corn stover is sourced, and manure is diverted from farms nearby. Avoided emissions from diverting food waste are allocated to the nearest landfill. More information on geographic allocation assumptions and specific location data is included in Section 8 of the Supporting Information.

## Results and Discussion

### Greenhouse Gas Emissions

The net GHG impact of the codigestion scenarios (Scenarios 2–5) is improved by substantial waste diversion and co-product offset credits (Figure 2). Waste diversion credits alone offset around half of the GHG emissions from baseline operations in each codigestion scenario. The largest co-product credits are associated with the energy-producing codigestion scenarios (Scenarios 2–3). In these cases, avoided emission credits result in more favorable net GHG footprints compared to the single-product base case (Scenario 1), which has a net GHG impact of 53 g of CO_2eq_ per MJ of ethanol (Figure 2). The simplest codigestion scenario, in which all biogas is used for electricity generation, has a life-cycle GHG impact of about 9 g of CO_2eq_ per MJ of ethanol. The benefits of offsetting grid electricity production in Scenario 2 are exceeded by those from offsetting diesel or natural gas in Scenarios 3A and 3B by about 66–75%. The only net-negative results are associated with biogas-to-RNG conversion. Using RNG as a feedstock to SMR for hydrogen production results in a GHG footprint of −9 g of CO_2eq_ emissions per MJ of ethanol. Using RNG for fleet fueling (in place of diesel) achieves a net GHG footprint of −26 g of CO_2eq_ per MJ of ethanol. Worth noting, however, is the fact that offset credits tied to fossil fuels are likely to decline as the grid decarbonizes and vehicles are electrified or otherwise transitioned away from fossil fuel use.

**Figure 2:**
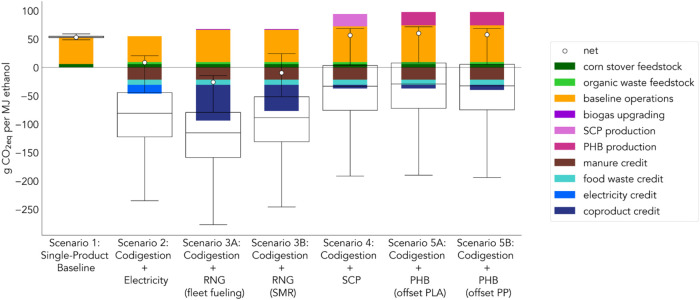
Net effects of each scenario on system-wide life-cycle greenhouse gas emissions, including offset credits for co-products and manure methane. The net result for each scenario is indicated by the white circular icon. The distribution of results from Monte Carlo simulations is shown by the box and whisker plots, where the boxes extend from the first to the third quartile of the data with a line at the median. The whiskers extend from the boxes to the farthest value that is within 1.5× the interquartile range of the boxes.

While positive contributors to emissions are not so different across Scenarios 1–3 (53–68 g of CO_2eq_/MJ ethanol), additional requirements for aerobic fermentation to produce either SCP or PHB make Scenarios 4–5 the most emission-intensive scenarios. They result in slightly higher GHG footprints (57–60 g of CO_2eq_ per MJ of ethanol) than the base case because co-product credits are modest and outweighed by the additional impacts from integrating aerobic fermentation. These results demonstrate that additional processing to create bioproducts may not be worthwhile from a GHG perspective. While these cases, like the other codigestion scenarios, benefit from manure and food waste diversion credits, biogas processing to produce biobased materials (vs energy resources) does not result in offset credits large enough to compete with the energy pathways for RNG. Prior research exploring this system found net-negative life-cycle GHG emissions for all codigestion scenarios examined, but this study provides a more extensive and conservative consideration of diversion and offset credits.^16^ The tabulated data for Figure 2 are provided in Table S11 of the Supporting Information.

The uncertainty associated with our results is substantial, as shown in the Monte Carlo simulation results indicated by the box and whisker plots in Figure 2. The key driver of variability and uncertainty is manure management and its impact on GHG emissions. This is reflected in the large negative range of possible results for the codigestion scenarios. This uncertainty will not impact the relative results across scenarios, provided that manure is sourced from the same location(s) in each case. However, if two different biorefineries corresponding to two of our scenarios elect to source manure from different locations with different counterfactual emissions, the uncertainty shown in Figure 2 is important to consider. Emissions from conventional swine and dairy manure management are variable due to factors outside a farm operator’s control, such as local climate conditions, and uncertain due to the lack of data on farm-level manure management practices. It is possible that diverting manure from on-farm management for codigestion will result in a deeply negative GHG intensity for ethanol produced if that manure is newly diverted from high-emission practices, such as lagoon storage.

Even with the uncertainty, our results show that there are several integration strategies that are very likely to offer near-term reductions in GHG emissions (Figure 2). The most promising biorefinery configuration is Scenario 3A, which involves codigestion and RNG production for use in fueling a fleet of trucks. As heavy-duty freight trucks are likely to be electrified more slowly than light- and medium-duty vehicles, this assumption should hold true in the near-term. Grid electricity, which is being rapidly decarbonized, is a major contributor to positive GHG emissions in the life-cycle for all scenarios except Scenario 2, so the GHG footprints of these scenarios are expected to decrease over time (Figure 3). As the impact of electricity lessens, the relative impact of other contributors (e.g., manure diversion credits) increases, which is why Scenarios 4–5, which currently are associated with higher GHG footprints than the base case (Figure 2), may become more favorable options in the near future (Figure 3). For Scenario 2, grid decarbonization will have little impact on the GHG footprint because net system-wide electricity consumption is relatively close to zero when taking into account electricity exports from the facility and upstream electricity consumption off-site (e.g., that which is embedded in consumed materials). Non-electricity offset and displacement credits are less certain in terms of their trends over time. If, for example, livestock operations were required to mitigate emissions from manure management, this would decrease offset credits for manure. However, these credits are generally not expected to change as considerably as the impacts associated with electricity. For additional results using NREL’s Cambium data for the “high renewables” U.S. electricity sector scenario, see Figure S2 in the Supporting Information.

**Figure 3:**
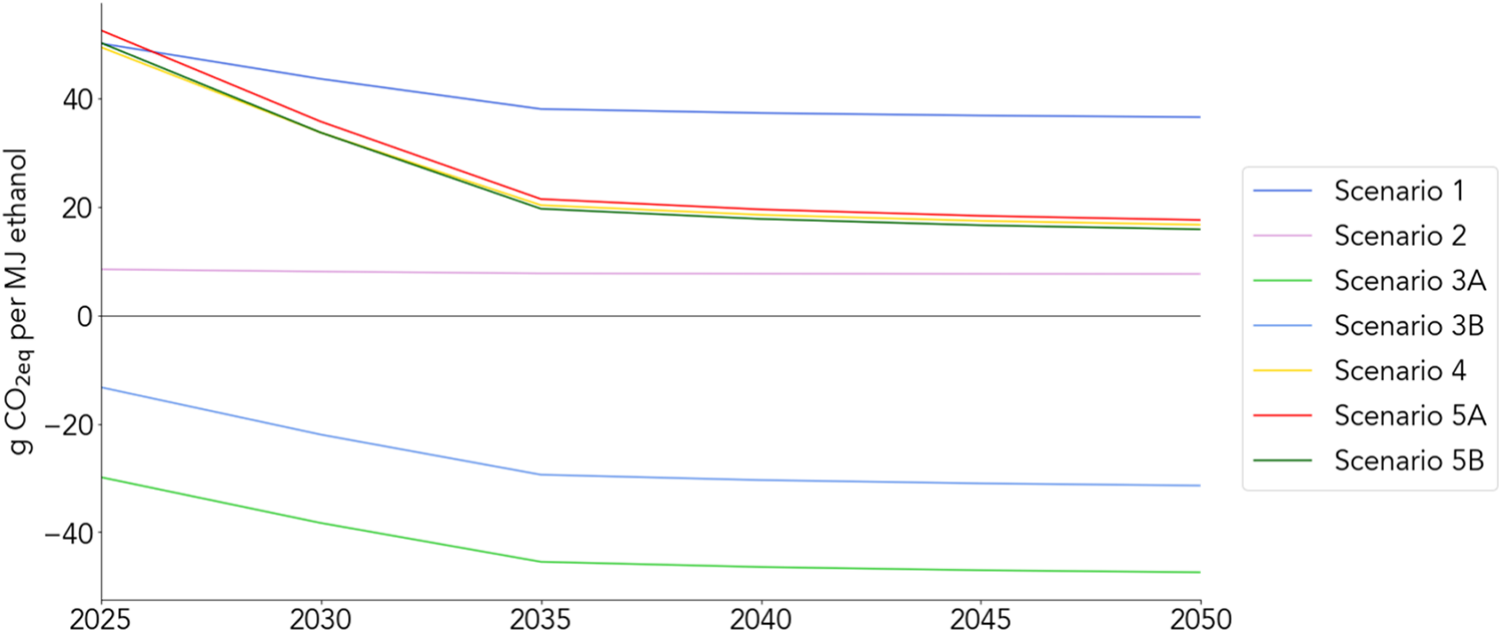
Greenhouse gas footprint forecasts based on NREL’s Cambium data for the “Mid-case” U.S. electricity sector scenario.

### Air Pollutant Emissions

In addition to GHG emissions, this study incorporates emissions of NH_3_, VOC, NO*_X_*, SO_2_, and PM_2.5_. The results for other gaseous pollutants are listed in Figure 4. Tabulated data for the plots in Figure 4 are provided in Tables S10–S14 of the Supporting Information. NH_3_ and VOCs stand out as being driven largely by manure diversion. While other contributors to life-cycle NH_3_ emissions are included, they are too small to be visible compared to counterfactual manure storage and field application. Emissions from manure storage can be reduced by using enclosed, negative-pressure environments and biofilters. Conversely, emissions from manure field application are hard to control. Diverting manure from field application will continue to provide offset credits to systems such as ours, even if conventional manure management is improved. As was the case for GHGs, NH_3_ emissions from manure management are highly uncertain, as shown by the large box plots in Figure 4a. This uncertainty could be reduced in part through more extensive NH_3_ measurement and monitoring campaigns. However, it is also dependent on the type of manure management strategy employed at individual farms, and this will change depending on where biorefineries source the manure. Fortunately, this uncertainty should not impact the study’s takeaways in terms of relative emission footprints and human health impacts if the goal is simply to compare across different biorefinery configurations.

**Figure 4:**
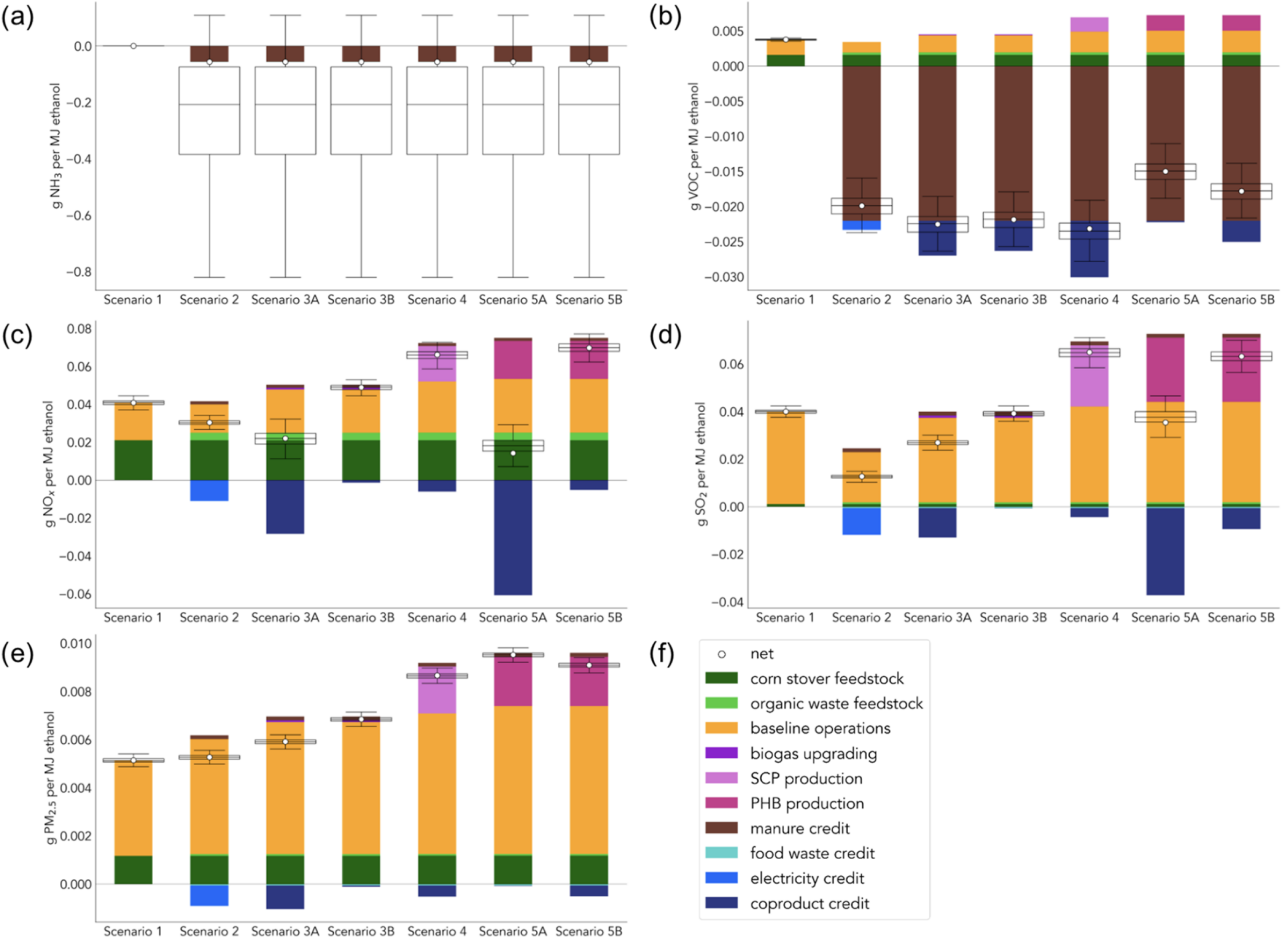
Net effects of each scenario on system-wide life-cycle air pollution emissions. The distribution of results from Monte Carlo simulations is shown by the box and whisker plots, where the boxes extend from the first to the third quartile of the data with a line at the median. The whiskers extend from the boxes to the farthest value that is within 1.5× the interquartile range of the boxes. (a) Life-cycle NH_3_ emissions. (b) Life-cycle VOC emissions. (c) Life-cycle NO_X_ emissions. (d) Life-cycle SO_2_ emissions. (e) Life-cycle PM_2.5_ emissions. (f) This legend applies to all plots (a–e) in this figure.

Regardless of the codigestion integration or biogas utilization pathway, the life-cycle emissions of NO_X_, SO_2_, and PM_2.5_ are net-positive in all scenarios. Unlike GHGs, VOCs, and NH_3_, these pollutants are driven almost entirely by combustion emissions. The narrow ranges reflected in the Monte Carlo simulations for these pollutants reflect limited data availability and calculations made using the specifics of the solid and gaseous fuels burned at these facilities. Ultimately, air permits will likely determine emissions rates at these facilities, and the ranges should be updated as more permit data becomes available for facilities with comparable fuel mixes. For NO*_X_* and SO_2_, the scenarios with codigestion for electricity and RNG fuel production (Scenarios 2 and 3A) emit less than the base case. However, codigestion is not always more favorable. Additional processing required in the SCP (Scenario 4) and PHB (Scenario 5) cases led to the highest positive NO*_X_* and SO_2_ emissions across all scenarios. In Scenario 5A, PHB is credited with offsetting PLA production, which is associated with relatively high NO*_X_* and SO_2_ emission factors.^52^ This co-product credit for offsetting PLA drives down the net-NO_*x*_ and net-SO_2_ impacts for this PHB scenario. However, if PHB offsets PP instead of PLA, then there are fewer NO*_X_* and SO_2_ benefits. PM_2.5_ emissions are net-positive in all scenarios and are lowest for the base case. Additional biogas processing in codigestion scenarios is associated with increased direct PM_2.5_ emissions, while the co-product credits are modest.

### Eutrophication and Acidification Potential

Some of the reported air emissions in the previous section also contribute to water quality and broader ecosystem impacts. Specifically, SO_2_ can contribute to acid rain, which impacts water bodies, soil, and human-made structures. NH_3_ emissions can deposit in water bodies where they contribute to eutrophication (deposit of excess nutrients, leading to an overabundance of algae and plants that deplete oxygen and kill other aquatic life). We selected marine eutrophication as the most relevant metric because this midpoint is dependent on nitrogen loading in water bodies, ultimately leading to runoff to oceans, and nitrogen deposition from NH_3_ emissions, which varies considerably across our scenarios.^57^ In contrast, it is unclear how much our scenarios will impact the fate of phosphorus, which drives freshwater eutrophication.^60^ Phosphorus is present in manure, but it remains in the residual solids that would ultimately be land-applied; therefore, it is unclear whether the use of anaerobic digestion would impact phosphorus loading in water bodies.

Net marine eutrophication and acidification potentials are reported for each scenario in Table 1. Detailed results broken down by pollutant are provided in the Supporting Information (Figures S3–S6). Most codigestion scenarios result in negative results for acidification potential because of avoided NH_3_ emissions associated with manure diversion, except Scenarios 4 and 5B, where the impact of relatively high life-cycle SO_2_ and NO*_X_* emissions outweighs the benefits of NH_3_ avoidance. All codigestion scenarios result in negative results for the marine eutrophication potential due to NH_3_ avoidance. Furthermore, reduced eutrophication potential due to manure diversion is likely underestimated because we conservatively assume that manure is not overapplied to lands in the counterfactual. We assume that urea fertilizer replaces manure for land application on a 1:1 basis with respect to nitrogen content. However, in actuality, farmers may overapply manure but are unlikely to overapply fertilizers to the same extent. In the case that manure is overapplied and manure diversion results in reduced nitrogen runoff, the avoided marine eutrophication potential is more substantial.

**Table 1:** Net Effect of Each Scenario on System-Wide Life-cycle Marine Eutrophication and Acidification Potential

	Life-cycle marine eutrophication potential (mg N-eq. per MJ ethanol)			
	Iowa	California	US average	life-cycle acidification potential (mg SO_2_-eq. per MJ ethanol)
scenario 1	1.18	0.91	1.17	70.61
scenario 2	–2.52	–2.81	–3.79	–68.14
scenario 3A	–2.76	–3.00	–4.03	–59.83
scenario 3B	–1.99	–2.41	–3.27	–28.78
scenario 4	–1.49	–2.03	–2.78	9.11
scenario 5A	–2.98	–3.17	–4.25	–56.08
scenario 5B	–1.39	–1.95	–2.68	10.57

### External Costs and Impacts on Local Populations

GHG and local air pollution emissions are normalized by cost in 2020 USD per gal of ethanol in Figure 5 to compare the external cost from each biorefinery configuration. The highest total climate and local human health damages are associated with the base case, single-product configuration, followed closely by Scenario 5A (PHB offsetting PLA), Scenario 5B (PHB offsetting PP), and Scenario 4 (SCP). The relative magnitude of climate damages and human health impacts varies by scenario. Net changes in human health as a result of air pollution exceed the effects of GHG emissions in Scenario 2 (codigestion with electricity generation) and Scenario 3B (codigestion with RNG production for SMR). Across all other scenarios, net effects on climate damages exceed local health impacts. The most favorable configuration from a local external cost perspective is codigestion with RNG production, which yielded the net-negative local external cost results. Of modeled RNG utilization pathways, fleet fueling has more local external benefits than SMR for hydrogen production because the RNG can be used to reduce emissions from diesel combustion in local trucks. When drawing conclusions from these results, it is important to note that only the local air pollution damages are included in the external cost analysis, while all life-cycle GHG emissions are considered. If nonlocal air pollution is emitted or avoided in densely populated areas, the share of total external costs from air pollution relative to GHG emissions may be greater.

**Figure 5:**
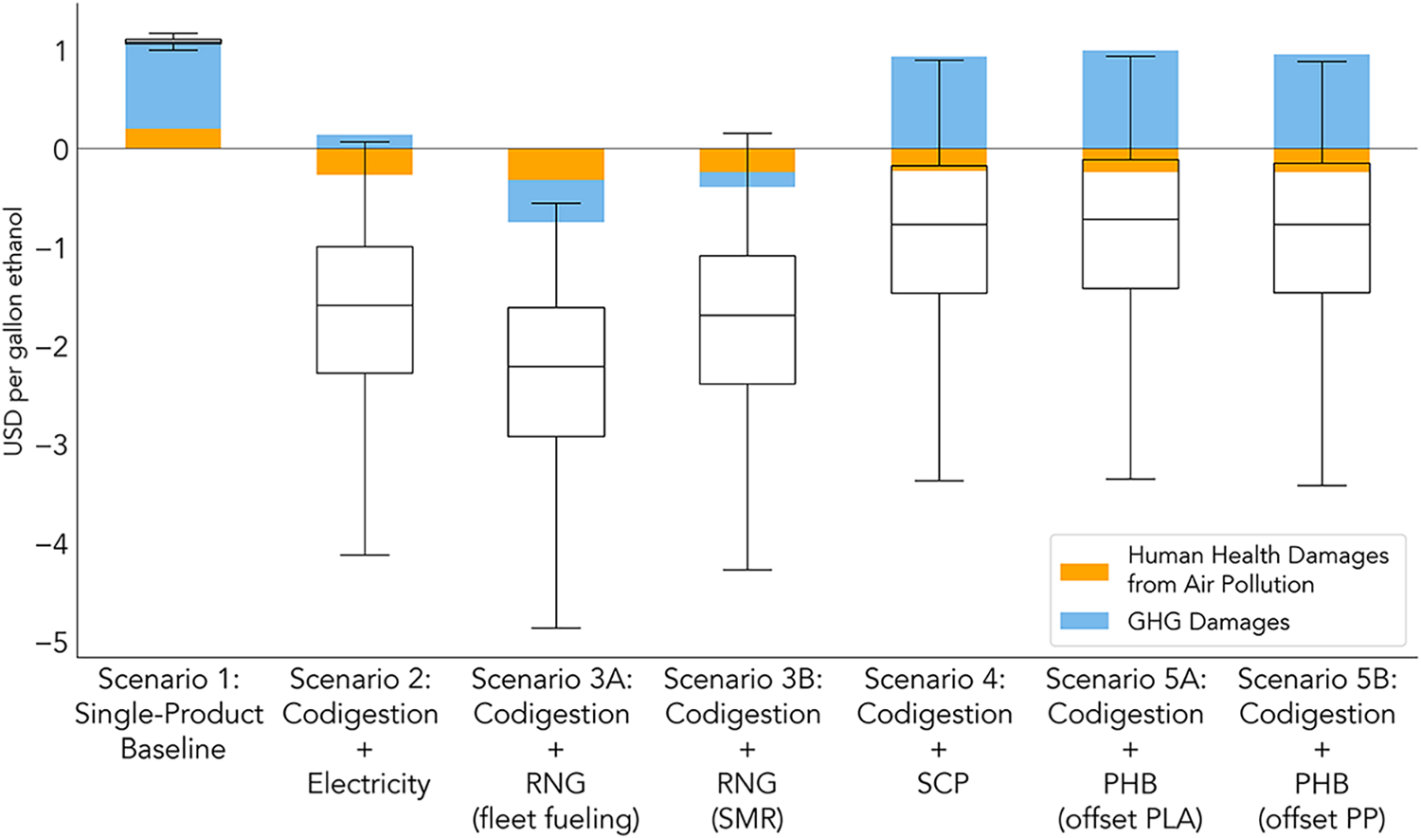
Farm-to-gate external costs from life-cycle GHG and local air pollution emissions. This figure shows local external cost results per gallon of ethanol (equivalent to 88.7 MJ ethanol) produced using ISRM and the LePeule function to calculate human health damages from air pollution for a hypothetical facility in Iowa. Other damages are calculated based on a monetary value of $185 USD per tonne of CO_2eq_. The box and whisker plots demonstrate the variability of cost results based on the Monte Carlo simulations of life-cycle GHG emissions (Figure 2). Because of the complexity of the ISRM model, Monte Carlo results for non-GHG air pollutants are not incorporated into the external cost analysis. Refer to the Supporting Information (Table S20 and Figure S7) for ISRM results using the Krewski function and IRSM results for California.

The results in Figure 5 provide strong evidence that air pollution impacts should not be ignored, even if a high value is placed on GHG mitigation. Organic waste diversion can meaningfully improve local air quality if facilities manage it responsibly. Integrating codigestion reduces emissions of harmful air pollutants, including NH_3_, VOCs, NO*_X_*, and SO_2_, relative to the base case scenario. The primary cause of these benefits is organic waste diversion, particularly manure, from conventional management practices. Diverting manure from long-term storage and land application to an enclosed digester may reduce burdens on local populations impacted by odor and air pollution from confined animal feeding operations. While manure provides benefits to agriculture, the equivalent urea fertilizer use has fewer NH_3_ and VOC emissions. However, diverting manure may also shift burdens from one local population to another if manure is trucked into a biorefinery from elsewhere, so these benefits may not be evenly experienced. There has also been some concern expressed about the potential of financial incentives for anaerobic digestion of manure to increase consolidation and the expansion of confined animal feeding operations.^61^ Ultimately, our analysis suggests that the net impact on local populations from manure diversion is worthy of further exploration, particularly in terms of its potential to reduce NH_3_ emissions and thus the level of secondary PM formation.

Co-products from improved biogas yields in codigestion scenarios provide a second opportunity for reducing the level of local air pollution and associated external costs. For instance, in Scenario 3A, local diesel transportation emissions are offset by RNG, which emits fewer GHGs, VOCs, NO*_X_*, and PM_2.5_ emissions when combusted. While there is uncertainty regarding some of the offsets modeled in this analysis, our results show opportunities to reduce all considered pollutant types except for direct PM_2.5_ with a multi-output facility configuration compared to the base case. Not all of these co-product benefits are reflected in the external cost results, because they cannot be attributed to the local region. Electricity consumption or credits can have an impact on local air pollution, but further modeling of power plants and demand response is required to accurately assess where associated air pollution impacts may occur. While producing SCP and/or PHB appears to have significant VOC, SO_2_, and/or NO*_X_* benefits from the life-cycle perspective, these benefits do not necessarily occur in the biorefinery region. Because there is substantial uncertainty regarding where these emissions are avoided, we do not include them in the local external cost analysis.

The integration of codigestion with biorefineries has potential not only as a mechanism for reducing GHG emissions but also as a means of reducing pollution from manure management and improving local air quality. All codigestion scenarios provide notable air quality benefits for the local population surrounding the biorefinery, primarily by avoiding NH_3_ and VOC emissions from conventional manure management. While the results presented in Figure 5 are specific to Iowa, we expect the local air quality benefits of manure diversion to be notable regardless of region, because conventional manure management is NH_3_-intensive. For instance, this conclusion remains true when considering a biorefinery in California (Figure S7). While in many cases establishing new industrial facilities poses risks and air pollution burdens to the surrounding population, carefully designed, integrated biorefineries can provide real benefits beyond reducing GHGs, eutrophication potential, and acidification potential and improve conditions impacting human health.

## Supplementary Material



## Notes

Additional methodology details, tabulated result data, and additional result figures (PDF)
