# Supplementary material for: Life-Cycle Emissions and Human Health Implications of Multi-Input, Multi-Output Biorefineries
Source: Environ Sci Technol. 2025 Aug 28;59(35):18562–72. doi: 10.1021/acs.est.4c12920 (PMC12424173; doi:10.1021/acs.est.4c12920)
Supplement: Supplementary file 1 [file es4c12920_si_001.pdf]

Supporting Information

Life-Cycle Emissions and Human Health Implications of Multi-Input, Multi-Output Biorefineries

Sarah L. Nordahl[1,2,3], Melissa Moore[3,4,5], Nawa R. Baral[3,4], Wilson McNeil[1,2], Yan Wang[4,5], and Corinne D. Scown[1,3,4,5*]

[1] Energy Technologies Area, Lawrence Berkeley National Laboratory, 1 Cyclotron Road, Berkeley, CA 94720, USA
[2] Department of Civil and Environmental Engineering, University of California, Berkeley, Berkeley, CA 94720, USA
[3] Joint BioEnergy Institute, 5885 Hollis Street, Emeryville, CA 94608, USA
[4] Biosciences Area, Lawrence Berkeley National Laboratory, 1 Cyclotron Road, Berkeley, CA 94720 United States
[5] Energy & Biosciences Institute, University of California, Berkeley, Berkeley, CA 94720, USA
*Email: cdscown@lbl.gov

Summary:
36 pages
7 figures
20 tables

I. Scenarios: Data and Assumptions

This section includes figures, data and assumptions that are scenario or study-specific. Facility designs and associated process data is from Wang et al. 2023. Figure S1 and associated data in Tables S1-2 are reprinted with permission from:

> Wang, Y.; Baral, N. R.; Yang, M.; Scown, C. D. Co-Processing Agricultural Residues and Wet Organic Waste Can Produce Lower-Cost Carbon-Negative Fuels and Bioplastics. *Environ. Sci. Technol.* **2023**, *57*, 2958–2969. American Chemical Society.

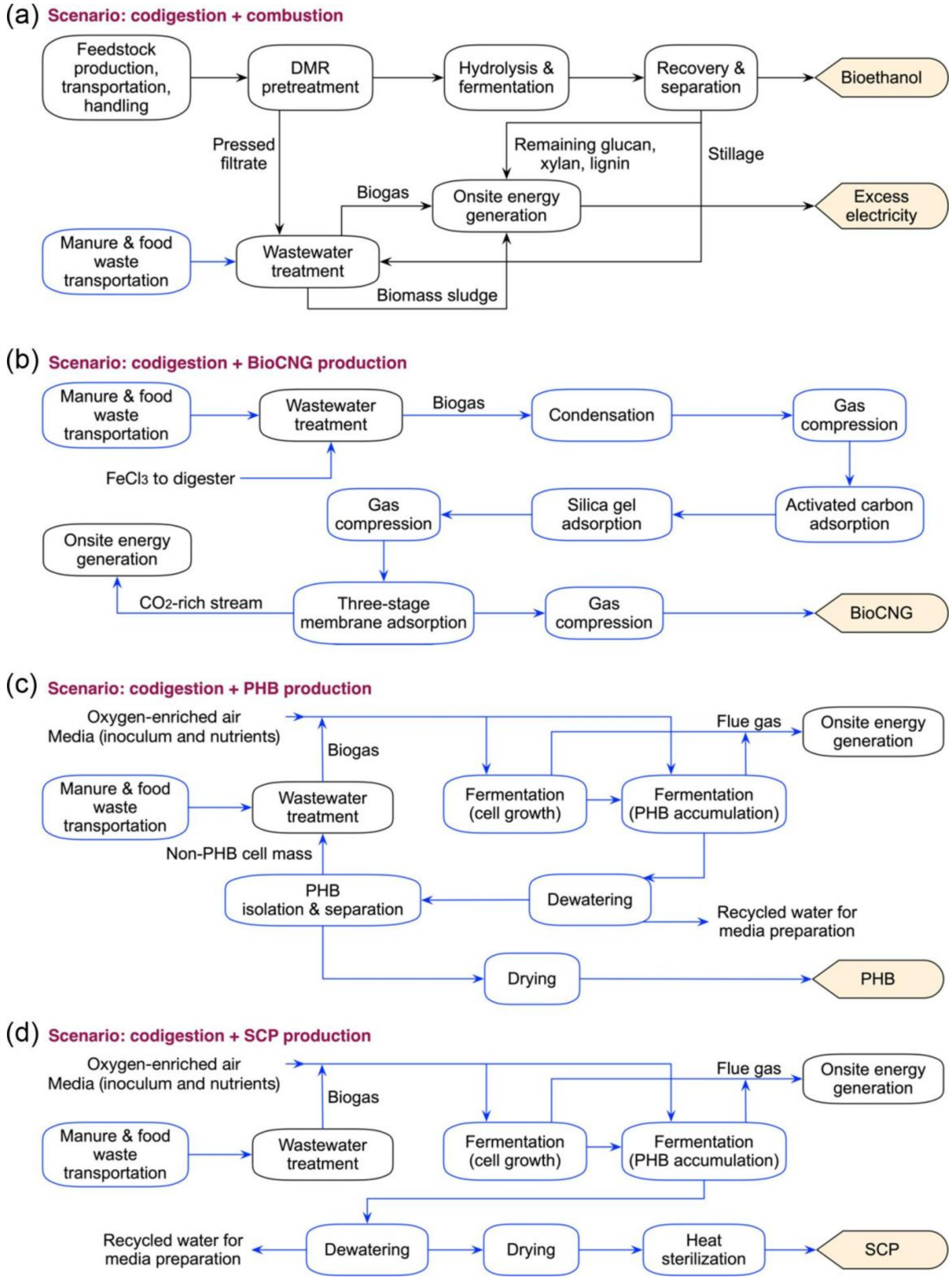

Figure S1. Detailed Flow Diagrams for Each Codigestion Biorefinery Facility Configuration Reproduced from Wang et al. 2023[1]

Part (a) corresponds to Scenario 2, part (b) corresponds to Scenario 3, part (c) corresponds to Scenario 5 and part (d) corresponds to Scenario 4. “BioCNG” refers to RNG. Reproduced from Wang et al. 2023.[1] Copyright 2023 American Chemical Society.

Table S1. Input and Output Data by Scenario from Wang et al. 2023[1]

Stage	Requirements	Units (per MJ bioEtOH)	Scenario				
			Scenario 1: Baseline	Scenario 2: Codig + Elec	Scenario 3: Codig + CNG	Scenario 4: Codig + SCP	Scenario 5: Codig + PHB
**Corn stover feedstock (Inputs)**							
	Corn stover	dry MT	1.29E-04	1.29E-04	1.29E-04	1.29E-04	1.29E-04
	Grid Electricity	kWh	1.15E-04	–	1.43E-04	2.19E-04	2.19E-04
**Organic waste feedstock (Inputs)**							
	Food waste	kg	–	2.58E-02	2.58E-02	2.58E-02	2.58E-02
	Cattle manure	kg	–	7.08E-02	7.08E-02	7.08E-02	7.08E-02
	Hog manure	kg	–	2.96E-01	2.96E-01	2.96E-01	2.96E-01
	Diesel (swine manure transportation)	L	–	8.30E-04	8.30E-04	8.30E-04	8.30E-04
	Diesel (cattle manure transportation)	L	–	1.98E-04	1.98E-04	1.98E-04	1.98E-04
**Pretreatment (Inputs)**							
	Grid Electricity	kWh	9.81E-03	0.00E+00	1.22E-02	1.86E-02	1.87E-02
	Hydrolase	kg	8.87E-04	8.87E-04	8.87E-04	8.87E-04	8.87E-04
	Sodium hydroxide	kg	6.44E-03	6.44E-03	6.44E-03	6.44E-03	6.44E-03
**Fermentation (Inputs)**							
	Grid Electricity	kWh	4.33E-04	–	5.37E-04	8.22E-04	8.26E-04
	Corn steep liquor (CSL)	kg	1.81E-03	1.81E-03	1.81E-03	1.81E-03	1.81E-03
	Diammonium phosphate (DAP)	kg	2.01E-04	2.01E-04	2.01E-04	2.01E-04	2.01E-04
	Dry yeast/inoculum	kg	4.59E-09	4.59E-09	4.59E-09	4.59E-09	4.59E-09
**Ethanol recovery (Inputs)**							
	Electricity	kWh	1.56E-03	0.00E+00	1.93E-03	2.96E-03	2.97E-03
**Wastewater treatment (Inputs)**							
	Electricity	kWh	2.72E-03	0.00E+00	5.48E-03	8.38E-03	1.09E-02
	Sodium hydroxide	kg	3.39E-06	3.39E-06	3.39E-06	3.39E-06	3.39E-06
	Glucose	kg	5.52E-06	5.52E-06	5.52E-06	5.52E-06	5.52E-06
**Onsite energy production (Inputs)**							
	Electricity	kWh	6.23E-04	0.00E+00	8.17E-04	1.30E-03	1.31E-03
	Lime	kg	1.73E-04	3.80E-04	2.82E-04	2.87E-04	3.01E-04
**Utilities (Inputs)**							
	Electricity	kWh	1.61E-03	0.00E+00	2.56E-03	3.69E-03	4.83E-03
**Biogas upgrading (Inputs)**							
	Electricity	kWh	--	--	2.76E-03	--	--
	Ferric chloride	kg	--	--	1.82E-04	--	--
	Activated carbon	kg	--	--	2.90E-07	--	--
	Silica gel	kg	--	--	8.50E-08	--	--
**PHB production (Inputs)**							
	Electricity	kWh	--	--	--	--	4.19E-02
	KH2PO4	kg	--	--	--	--	1.03E-03

	NH4OH	kg	--	--	--	--	1.07E-03
	Salts (magnesium sulfate)	kg	--	--	--	--	3.14E-04
	H2O2	kg	--	--	--	--	1.45E-03
**SCP production (Inputs)**							
	Electricity	kWh	--	--	--	3.81E-02	--
	KH2PO4	kg	--	--	--	1.03E-03	--
	NH4OH	kg	--	--	--	1.07E-03	--
	Salts (magnesium sulfate)	kg	--	--	--	3.14E-04	--
**Waste (Outputs)**							
	Ash waste	kg	2.17E-03	2.17E-03	2.27E-03	2.17E-03	2.17E-03
	Solid waste	kg	5.39E-03	8.81E-03	8.93E-03	8.81E-03	8.81E-03
	CaSO4 waste	kg	2.85E-04	6.26E-04	4.64E-04	4.73E-04	4.95E-04
	Trucking	tonne-km	6.94E-04	1.03E-03	1.03E-03	1.01E-03	1.02E-03
**Electricity generation (Outputs)**							
	Electricity	kWh	–	3.23E-02	--	--	--
**Coproduct generation (Outputs)**							
	CNG produced	kg	--	--	1.43E-02	--	--
	PHB produced	kg	--	--	--	--	4.94E-03
	SCP produced	kg	--	--	--	8.20E-03	--

Table S2. Process Data for Assessing Direct Facility Emissions from Wang et al. 2023[1]

Stage with Direct Emissions	Emitting Process	Units	Scenario 1: Baseline	Scenario 2: Codigestion + Electricity	Scenario 3: Codigestion + RNG	Scenario 4: Codigestion + SCP	Scenario 5: Codigestion + PHB
Wastewater treatment	SO_2 generation in flue gas directly emitted to atmosphere	kg of SO_2 per hour	1.43E+01	6.50E+00	1.06E+01	1.09E+01	1.13E+01
Wastewater treatment	NH_3 generation in biogas	tonnes of NH_3 per day	8.31E-02	1.94E-01	1.94E-01	1.94E-01	1.94E-01
Wastewater treatment	Methane generation in biogas	tonnes of CH_4 per day	1.19E+02	2.33E+02	2.33E+02	2.33E+02	2.33E+02
Wastewater treatment	H_2S generation in biogas	tonnes of H_2S per day	4.60E-01	9.89E-01	9.89E-01	9.89E-01	1.18E+00
Onsite energy production	Biogas combustion	m^3 biogas input per day	3.73E+05	7.16E+05	3.87E+05	6.96E+05	2.66E+05
Onsite energy production	Solids combustion	tonnes combusted per day	7.05E+02	1.06E+03	1.08E+03	1.09E+03	1.16E+03

Table S3. LCA Modeling Assumptions by Stage

Stage/Plot Category	Modeling assumptions
**Corn stover feedstock**	● Modeling includes impacts from corn stover collection, transportation and handling.<br>● For corn stover collection and transportation, only impacts from additional farming requirements (increased fertilizer and diesel use) for corn stover collection and transportation are considered.<br>● Electricity is used for on-site corn stover handling.
**Organic waste feedstock**	● Modeling includes impacts from waste diversion and transportation.<br>● Manure and food wastes are considered burden-free (no upstream emissions burden besides transportation).<br>● Organic waste inputs are used to evaluate diversion credits (see Table S3).<br>● Modeling assumes diesel trucking for transportation.
**Pretreatment**	● Modeling includes impacts from energy and material use.<br>● Because insufficient LCA data for hydrolase is available, cellulase is used as a proxy for hydrolase.
**Fermentation**	● Modeling includes impacts from energy and material use.<br>● Inoculum use is assumed to have a negligible impact because of relatively small use rate.
**Ethanol recovery**	● Modeling includes impacts from electricity use.
**Wastewater treatment**	● Modeling includes impacts from energy and material use (data in Table S1), and direct emissions (data in Table S2).
**Onsite energy production**	● Modeling includes impacts from energy and material use.
**Utilities**	● Modeling includes impacts from electricity use.
**Biogas upgrading**	● Modeling includes impacts from energy and material use.
**PHB production**	● Modeling includes impacts from energy and material use.<br>● Langbeinite is used as a proxy for magnesium sulfate salts.
**SCP production**	● Modeling includes impacts from energy and material use.<br>● Langbeinite is used as a proxy for magnesium sulfate salts.
**Waste**	● Modeling includes impacts from waste transportation.<br>● Impacts from ash, solid waste and $CaSO_4$ waste treatment are assumed negligible.
**Electricity generation**	● Modeling assumes surplus electricity is exported to the grid to provide offset credits. See Table S4.
**Coproduct generation**	● Non-electricity coproducts include CNG, SCP and PHB. All provide offset credits. See Table S4.

Table S4. Summary of LCA Modeling Assumptions for Offset and Diversion Credits

Category	Assumptions
**Food waste credit**	• Food waste is assumed to be diverted from landfilling.
**Manure credit**	• Impacts include avoided emissions and induced fertilizer use.<br>• Both swine and cattle manure are assumed to be diverted from outdoor storage and field application. For dairy manure, we use CH_4, N_2O, and NH_3 emission factors from Amon et al. (2006) based on dairy manure during slurry tank storage and field application to grassland. For swine manure, we use CH_4, N_2O, and NH_3 emission factors for deep pit storage and application to paddy fields from Wang et al. (2017). See Table S5 for emission factor data.<br>• An average VOC emission factor for manure treatment is used for estimating avoided VOC emissions (see Table S5).<br>• Manure diversion is assumed to induce urea demand based on nitrogen content. In addition to upstream impacts from urea production, differences in N_2O and NH_3 emissions from urea application vs. manure application are considered (see Table S5).
**Electricity credit**	• Surplus electricity is exported to the grid and assumed to offset emissions from the Midwest Reliability Organization (MRO) grid region in the U.S.
**Coproduct credit**	• Biogas can be upgraded to create non-electricity coproducts. This study considers 3 such coproducts: bio CNG, SCP and PHB.<br>• Scenario 3A and 3B consider bio CNG production varying CNG use case. Scenario 3A assumes CNG will be used to fuel truck fleets and offsets diesel. Scenario 3B assumes CNG will be used as a feedstock to steam methane reformation (SMR) for hydrogen production offsetting natural gas production and use.<br>• Scenario 4 assumes SCP will offset demand for soybean meal, based on protein content, and avoid associated production emissions.<br>• Scenario 5A assumes PHB will offset demand for PLA, the primary bioplastic on the market, on a 1:1 mass basis and avoid associated production emissions.<br>• Scenario 5B assumes PHB will offset demand for polypropylene on a 1:1 mass basis and avoid associated production emissions.

II. LCA Model

Table S5. LCA Data: Emission Factors

All values are in units of kg of pollutant (given by column name) per unit indicated at the end of the unit process name. These are not necessarily life-cycle emission factors and only definitely include direct emission impacts (in several cases, this means fugitive emissions associated with fuel combustion); full life-cycle impacts must be assessed through the model using this data along with IO data from Table S6. When possible, GHG emission factors are separated by pollutant type; in cases where this is not possible, total GHG impact in CO_2 equivalence is given by the CO_2 column while the CH_4 and N_2O columns are marked with zeros. Zero entries can indicate no direct emissions, negligible direct emissions or unknown direct emissions.

Unit process	CO_2	CH_4	N_2O	NH_3	$PM_{2.5}$	SO_2	NO_x	VOCs	Source(s)
atrazine.kg	8.71E+00	1.56E-04	1.25E-04	0.00E+00	7.80E-04	2.27E-04	2.85E-02	0.00E+00	[2]
glyphosate.kg	0.00E+00	0.00E+00	0.00E+00	0.00E+00	0.00E+00	0.00E+00	0.00E+00	0.00E+00	NA
insecticide.kg	2.31E+01	4.06E-02	3.63E-04	0.00E+00	0.00E+00	0.00E+00	0.00E+00	0.00E+00	[3]
cellulase.kg	7.17E+00	1.60E-02	4.04E-03	0.00E+00	0.00E+00	0.00E+00	0.00E+00	0.00E+00	[3]
lime.kg	1.11E+00	9.54E-07	9.86E-08	0.00E+00	1.16E-05	5.56E-05	2.34E-03	0.00E+00	[2]
CaCO3.kg	1.61E-03	5.15E-08	3.19E-08	0.00E+00	1.04E-05	2.90E-07	6.48E-04	0.00E+00	[2]
NaOH.kg	4.68E-01	8.09E-06	4.32E-06	0.00E+00	0.00E+00	0.00E+00	0.00E+00	0.00E+00	[2]
csl.kg	1.24E-01	2.21E-06	1.15E-06	0.00E+00	7.31E-06	5.60E-07	8.07E-05	5.29E-06	[2]
dap.kg	1.38E-02	2.46E-07	2.46E-07	0.00E+00	0.00E+00	0.00E+00	0.00E+00	0.00E+00	[2]
glucose.kg	8.90E-03	1.59E-07	8.25E-08	0.00E+00	0.00E+00	0.00E+00	0.00E+00	0.00E+00	[2]
corn_starch.kg	5.02E-01	1.39E-03	8.83E-04	0.00E+00	0.00E+00	0.00E+00	0.00E+00	0.00E+00	[3]
K2O.kg	4.50E-01	8.91E-04	7.64E-06	0.00E+00	1.96E-05	6.40E-07	1.16E-03	0.00E+00	[3]
ammonia.conventional.kg	1.69E+00	9.41E-06	9.41E-06	0.00E+00	0.00E+00	2.88E-05	0.00E+00	0.00E+00	[2,4]
n.kg	2.00E-01	3.81E-04	3.09E-06	0.00E+00	2.28E-04	1.33E-05	8.13E-03	0.00E+00	[3]
urea.kg	3.57E-01	1.11E-03	9.71E-06	0.00E+00	0.00E+00	0.00E+00	0.00E+00	0.00E+00	[3]
P2O5.kg	6.74E-02	1.29E-04	1.04E-06	0.00E+00	2.31E-05	3.55E-07	1.62E-03	0.00E+00	[3]
glycerin.kg	1.91E-01	4.90E-04	2.69E-06	0.00E+00	0.00E+00	0.00E+00	0.00E+00	0.00E+00	[3]
NaCl.kg	1.80E-01	4.00E-04	3.38E-06	0.00E+00	0.00E+00	0.00E+00	0.00E+00	0.00E+00	[3]
coal.MJ	6.97E-04	1.39E-04	8.73E-09	0.00E+00	2.16E-08	8.05E-09	1.04E-06	0.00E+00	[3]
diesel.MJ	5.09E-03	3.93E-08	2.02E-07	0.00E+00	4.60E-07	1.59E-05	4.08E-06	0.00E+00	[2]
RFO.MJ	4.00E-03	8.74E-06	6.83E-08	0.00E+00	8.30E-08	3.72E-09	2.07E-06	0.00E+00	[5]
refgas.MJ	7.06E-02	0.00E+00	0.00E+00	0.00E+00	0.00E+00	0.00E+00	0.00E+00	0.00E+00	[3]
crudeoil.MJ	2.62E-03	8.24E-05	3.12E-08	0.00E+00	4.90E-08	3.87E-09	1.55E-06	0.00E+00	[5]
electricity.US.kWh	4.14E-01	4.40E-05	7.00E-06	0.00E+00	2.40E-05	2.48E-04	2.38E-04	7.00E-06	[6]
electricity.MRO.kWh	4.13E-01	4.04E-05	5.90E-06	0.00E+00	2.40E-05	3.23E-04	3.16E-04	7.00E-06	[6,7]
gasoline.MJ	3.56E-03	3.48E-05	1.32E-07	0.00E+00	1.89E-07	5.70E-05	1.68E-05	0.00E+00	[5]
H2.kg	1.06E+01	5.98E-02	4.00E-05	0.00E+00	2.00E-03	9.50E-03	1.23E-02	0.00E+00	[8]
Naturalgas.<br>conventional.MJ	4.06E-03	8.70E-05	2.60E-08	0.00E+00	6.00E-08	4.74E-09	1.89E-06	6.10E-06	[2]
Naturalgas.<br>shale.MJ	3.80E-03	9.19E-05	2.59E-08	0.00E+00	6.00E-08	4.74E-09	1.89E-06	6.10E-06	[2]
naturalgas_select.MJ	0.00E+00	0.00E+00	0.00E+00	0.00E+00	0.00E+00	0.00E+00	0.00E+00	0.00E+00	NA;<br>Selection<br>variable with

									IO references to shale and conventional production shares
silica.kg	0.00E+00	0.00E+00	0.00E+00	0.00E+00	0.00E+00	0.00E+00	0.00E+00	0.00E+00	Assume negligible for this system
flatbedtruck.mt_km	1.24E-01	0.00E+00	0.00E+00	0.00E+00	2.82E-06	2.82E-06	2.54E-05	0.00E+00	9
tankertruck.mt_km	8.46E-02	0.00E+00	0.00E+00	0.00E+00	1.93E-06	1.93E-06	1.74E-05	0.00E+00	9
gaspipeline.mt_km	0.00E+00	0.00E+00	0.00E+00	0.00E+00	0.00E+00	0.00E+00	0.00E+00	0.00E+00	8,10
liquidpipeline.mt_km	0.00E+00	0.00E+00	0.00E+00	0.00E+00	0.00E+00	0.00E+00	0.00E+00	0.00E+00	NA
rail.mt_km	1.86E-02	0.00E+00	0.00E+00	0.00E+00	4.25E-07	4.25E-07	3.83E-06	0.00E+00	9
barge.mt_km	2.22E-02	0.00E+00	0.00E+00	0.00E+00	5.08E-07	5.08E-07	4.57E-06	0.00E+00	9
marinetanker.mt_km	6.91E-03	8.02E-08	0.00E+00	0.00E+00	8.99E-07	4.50E-07	1.35E-05	0.00E+00	9
corn.bushel	6.70E-01	0.00E+00	0.00E+00	0.00E+00	0.00E+00	0.00E+00	0.00E+00	0.00E+00	3
butane.MJ	5.16E-03	2.24E-07	4.35E-08	0.00E+00	0.00E+00	0.00E+00	0.00E+00	0.00E+00	11
lpg.kg	5.22E-01	1.45E-03	8.28E-06	0.00E+00	0.00E+00	0.00E+00	0.00E+00	0.00E+00	11
phosphoric.acid.kg	1.01E+00	2.16E-03	2.44E-05	0.00E+00	8.20E-04	1.10E-02	4.47E-03	3.28E-04	11
corn.stover.kg	1.92E-02	4.37E-07	4.98E-07	0.00E+00	7.31E-06	1.12E-07	1.07E-04	9.62E-06	2
landfill.foodwaste.wet_kg	0.00E+00	1.30E-02	0.00E+00	1.41E-06	2.68E-06	2.74E-05	4.07E-07	4.18E-08	12,13
ferric.chloride.kg	1.95E-01	0.00E+00	0.00E+00	0.00E+00	0.00E+00	0.00E+00	0.00E+00	0.00E+00	14
Na.brine.kg	5.47E-02	1.19E-06	6.20E-07	0.00E+00	0.00E+00	0.00E+00	0.00E+00	0.00E+00	2
activated.carbon.kg	5.29E+00	5.78E-04	5.78E-05	0.00E+00	1.16E-03	2.89E-02	1.16E-02	9.94E-05	15,16
KH2PO4.kg	0.00E+00	0.00E+00	0.00E+00	0.00E+00	0.00E+00	0.00E+00	0.00E+00	0.00E+00	6
KOH.kg	1.73E-01	3.09E-06	1.78E-06	0.00E+00	1.70E-05	1.04E-06	1.87E-04	4.65E-06	6
KCl.kg	1.82E-01	3.28E-06	2.92E-06	0.00E+00	1.82E-05	3.43E-04	1.97E-04	7.81E-06	6
NH4OH.kg	0.00E+00	0.00E+00	0.00E+00	0.00E+00	0.00E+00	0.00E+00	0.00E+00	0.00E+00	6
langbeinite.kg	1.73E-01	3.09E-06	1.78E-06	0.00E+00	1.70E-05	1.04E-06	1.87E-04	4.65E-06	6
swine.manure.storage.kg	0.00E+00	2.02E-04	1.69E-04	2.37E-04	0.00E+00	0.00E+00	0.00E+00	6.10E-05	17,18
dairy.manure.storage.kg	0.00E+00	4.05E-03	2.02E-05	4.10E-05	0.00E+00	0.00E+00	0.00E+00	6.10E-05	18–20
Swine.manure.field. application.kg	0.00E+00	1.25E-03	1.50E-07	4.36E-04	0.00E+00	0.00E+00	0.00E+00	0.00E+00	17,20
Dairy.manure.field. application.kg	0.00E+00	1.30E-06	3.80E-06	5.09E-04	0.00E+00	0.00E+00	0.00E+00	0.00E+00	19,20
Diesel.combust. heavydutytruck.MJ	7.39E-02	7.56E-07	1.01E-07	0.00E+00	8.80E-07	5.17E-07	9.63E-05	5.51E-06	6
PLA.kg	8.27E-01	1.31E-02	3.71E-04	3.95E-06	1.94E-06	7.40E-03	1.23E-02	4.32E-05	21
urea_application.kg	0.00E+00	0.00E+00	6.35E-03	3.84E-02	0.00E+00	0.00E+00	0.00E+00	0.00E+00	20,22
soybeanmeal.kg	2.36E-01	5.56E-04	8.27E-04	0.00E+00	3.53E-05	2.93E-04	4.83E-04	6.28E-04	6
CNG.combust. heavydutytruck.MJ	0.00E+00	1.80E-05	5.00E-06	0.00E+00	4.60E-07	5.60E-07	6.50E-05	1.60E-06	23
biogas_CHP.m3	0.00E+00	5.67E-03	-2.00E-06	0.00E+00	0.00E+00	1.32E-04	1.07E-04	4.50E-05	24
biomass_combust.kg	0.00E+00	0.00E+00	0.00E+00	0.00E+00	5.45E-02	0.00E+00	6.56E-03	1.59E-04	25
polypropylene.kg	9.14E-01	6.42E-03	1.56E-05	1.50E-06	6.87E-05	1.63E-03	7.75E-04	3.44E-04	26,27

Table S6. LCA Model: Input-Output Matrix Relationships

Our LCA model uses a physical units-based input-output matrix that is populated with life-cycle inventories for each unit process/product included. The relevant non-zero values are included in this table. Each unit process/product is listed with a unit. The value indicates the amount of the upstream/downstream requirement in its listed unit required to make 1 unit of the primary unit product/process. For unit processes from Table S5 that are not included in this table, there are either (1) no upstream/downstream impacts for that parameter or (2) insufficient input/output data is available and full life-cycle emissions are included in the emission factors in Table S5.

Unit process	Life-cycle requirements	Value	Units	Source
atrazine				
	diesel	4.41E+01	MJ/kg	2
	RFO	4.41E+01	MJ/kg	2
	electricity.US	6.94E+00	kWh/kg	2
	natural gas_select	3.38E+01	MJ/kg	2
	tanker truck	4.26E-01	mt_km/kg	3
	rail	1.26E+00	mt_km/kg	3
glyphosate				
	tankertruck	4.26E-01	mt_km/kg	3
	rail	1.26E+00	mt_km/kg	3
insecticide				
	diesel	1.53E+02	MJ/kg	3
	electricity.US	1.28E+01	kWh/kg	3
	naturalgas_select	5.86E+01	MJ/kg	3
	tankertruck	4.26E-01	mt_km/kg	3
	rail	1.26E+00	mt_km/kg	3
cellulase				
	csl	2.87E-01	kg/kg	3
	glucose	3.90E+00	kg/kg	3
	ammonia_conventional	1.90E-01	kg/kg	3
	glycerin	4.00E-01	kg/kg	3
	NaCl	2.00E-01	kg/kg	3
	electricity.MRO	1.06E+01	kWh/kg	3
	naturalgas_select	2.24E+00	MJ/kg	3
lime				
	CaCO3	1.88E+00	kg/kg	2
	coal	3.57E+00	MJ/kg	2
	diesel	7.43E-02	MJ/kg	2
	RFO	3.24E-02	MJ/kg	2
	electricity.US	5.70E-02	kWh/kg	2
	naturalgas_select	2.25E-01	MJ/kg	2
	tankertruck	1.93E-01	mt_km/kg	3
	lpg	4.19E-02	kg/kg	2

CaCO3				
	coal	3.66E-03	MJ/kg	2
	diesel	1.30E-02	MJ/kg	2
	RFO	1.64E-03	MJ/kg	2
	electricity.US	2.44E-04	kWh/kg	2
	gasoline	2.56E-03	MJ/kg	2
	naturalgas_select	1.22E+00	MJ/kg	2
	flatbedtruck	8.00E-02	mt_km/kg	3
NaOH				
	coal	6.01E-01	MJ/kg	2
	RFO	2.11E-02	MJ/kg	2
	electricity.US	1.67E+00	kWh/kg	2
	naturalgas_select	7.28E+00	MJ/kg	2
	tankertruck	4.26E-01	mt_km/kg	2
	rail	1.26E+00	mt_km/kg	2
	na_brine	5.83E+00	kg/kg	2
csl				
	electricity.MRO	6.46E-03	kWh/kg	6
	naturalgas_select	2.20E+00	MJ/kg	6
	corn	2.19E-01	bushel/kg	6
dap				
	ammonia_conventional	2.20E-01	kg/kg	2
	RFO	1.19E-01	MJ/kg	2
	electricity.US	3.02E-02	kWh/kg	2
	naturalgas_select	2.45E-01	MJ/kg	2
	phosphoric-acid	9.94E-01	kg/kg	2
glucose				
	NaOH	2.82E-06	kg/kg	2
	corn_starch	9.40E-01	kg/kg	2
	naturalgas_select	1.58E-01	MJ/kg	2
corn_starch				
	electricity.MRO	9.05E-02	kWh/kg	3
	naturalgas_select	4.02E+00	MJ/kg	3
	corn	6.89E-02	bushel/kg	3
K2O				
	diesel	2.40E+00	MJ/kg	3
	naturalgas_select	2.70E+00	MJ/kg	3
	tankertruck	4.26E-01	mt_km/kg	3

	rail	1.26E+00	mt_km/kg	3
Ammonia_ conventional				
	electricity.US	1.18E-01	kWh/kg	2
	naturalgas_select	3.31E+01	MJ/kg	2
	tankertruck	4.26E-01	mt_km/kg	3
	rail	1.26E+00	mt_km/kg	3
n (Nitrogen)				
	diesel	1.80E+00	MJ/kg	3
	naturalgas_select	4.40E+01	MJ/kg	3
	tankertruck	4.26E-01	mt_km/kg	3
	rail	1.26E+00	mt_km/kg	3
urea				
	electricity.US	1.29E+00	kWh/kg	2
	naturalgas_select	5.16E+00	MJ/kg	2
	tankertruck	4.26E-01	mt_km/kg	3
	rail	1.26E+00	mt_km/kg	3
P2O5				
	diesel	3.90E+00	MJ/kg	3
	naturalgas_select	1.50E+00	MJ/kg	3
	tankertruck	4.26E-01	mt_km/kg	3
	rail	1.26E+00	mt_km/kg	3
NaCl				
	RFO	1.28E-01	MJ/kg	3
	electricity.US	7.11E-02	kWh/kg	3
	naturalgas_select	7.91E-01	MJ/kg	3
coal				
	diesel	2.30E-03	MJ/MJ	3
	RFO	2.53E-04	MJ/MJ	3
	electricity.US	2.09E-03	kWh/MJ	3
	gasoline	1.89E-04	MJ/MJ	3
	naturalgas_select	5.82E-05	MJ/MJ	3
	flatbedtruck	2.55E-03	mt_km/MJ	3
	rail	5.34E-02	mt_km/MJ	3
	barge	2.24E-02	mt_km/MJ	3
diesel				
	RFO	3.11E-02	MJ/MJ	2
	refgas	5.81E-02	MJ/MJ	2
	crudeoil	1.00E+00	MJ/MJ	2
	electricity.US	8.93E-04	kWh/MJ	2
	H2	1.08E-04	kg/MJ	2

	naturalgas_select	5.19E-02	MJ/MJ	[2]
	tankertruck	3.53E-03	mt_km/MJ	[2]
	liquidpipeline	2.10E-02	mt_km/MJ	[2]
	butane	9.92E-05	MJ/MJ	[2]
RFO (residual fuel oil)				
	RFO	2.70E-02	MJ/MJ	[2]
	refgas	3.73E-02	MJ/MJ	[2]
	crudeoil	1.00E+00	MJ/MJ	[2]
	electricity.US	3.71E-04	kWh/MJ	[2]
	H2	1.20E-05	kg/MJ	[2]
	naturalgas_select	2.43E-02	MJ/MJ	[2]
	tankertruck	3.53E-03	mt_km/MJ	[2]
	liquidpipeline	2.10E-02	mt_km/MJ	[2]
	butane	7.20E-05	MJ/MJ	[2]
refgas				
	crudeoil	1.00E+00	MJ/MJ	NA
crudeoil				
	diesel	3.06E-03	MJ/MJ	[2]
	RFO	2.04E-04	MJ/MJ	[2]
	crudeoil	2.04E-04	MJ/MJ	[2]
	electricity.US	1.08E-03	kWh/MJ	[2]
	gasoline	4.08E-04	MJ/MJ	[2]
	naturalgas_select	1.26E-02	MJ/MJ	[2]
	tankertruck	5.49E-03	mt_km/MJ	[2]
	liquidpipeline	2.85E-02	mt_km/MJ	[2]
	rail	2.42E-02	mt_km/MJ	[2]
	barge	4.39E-03	mt_km/MJ	[2]
	marinetanker	1.10E-01	mt_km/MJ	[2]
electricity.MRO				
	coal	3.22E+00	MJ/kWh	[7,28]
	RFO	1.54E-02	MJ/kWh	[7,28]
	electricity.MRO	5.10E-02	kWh/kWh	[7,28]
	naturalgas_select	1.50E+00	MJ/kWh	[7,28]
gasoline				
	RFO	9.28E-02	MJ/MJ	[29]
	refgas	9.26E-02	MJ/MJ	[29]
	crudeoil	1.00E+00	MJ/MJ	[29]
	electricity.US	1.44E-02	kWh/MJ	[29]
	H2	5.26E-05	kg/MJ	[29]

	naturalgas_select	6.27E-02	MJ/MJ	29
	tankertruck	3.53E-03	mt_km/MJ	29
	liquidpipeline	2.10E-02	mt_km/MJ	29
	butane	6.44E-02	MJ/MJ	29
H2				
	electricity.US	2.69E-01	kWh/kg	8
	naturalgas_select	1.43E+02	MJ/kg	8
	gaspipeline	1.21E+00	mt_km/kg	8
silica				
	flatbedtruck	8.00E-02	mt_km/kg	General assumption (50 mi by truck)
	rail	8.00E-01	mt_km/kg	General assumption (50 mi by truck)
flatbedtruck				
	diesel	1.78E+00	MJ/mt_km	9
	flatbedtruck	2.50E-01	mt_km/mt_km	9
tankertruck				
	diesel	1.22E+00	MJ/mt_km	9
	tankertruck	2.50E-01	mt_km/mt_km	9
liquidpipeline				
	electricity.US	1.84E-02	kWh/mt_km	30
rail				
	diesel	2.68E-01	MJ/mt_km	9
	rail	2.50E-01	mt_km/mt_km	9
barge				
	diesel	3.20E-01	MJ/mt_km	9
	barge	2.50E-01	mt_km/mt_km	9
marinetanker				
	RFO	1.00E-01	MJ/mt_km	9
	marinetanker	2.50E-01	mt_km/mt_km	9
corn				
	atrazine	3.50E-03	kg/bushel	3
	glyphosate	3.50E-03	kg/bushel	3
	insecticide	6.00E-05	kg/bushel	3
	CaCO3	1.15E+00	kg/bushel	3
	K2O	1.51E-01	kg/bushel	3
	n	4.23E-01	kg/bushel	3
	P2O5	1.46E-01	kg/bushel	3
	diesel	9.65E+00	MJ/bushel	3
	flatbedtruck	2.00E+00	mt_km/bushel	3
butane				

	naturalgas_select	1.00E+00	MJ/MJ	NA
NaOH				
	coal	6.01E-01	MJ/kg	[2]
	RFO	2.11E-02	MJ/kg	[2]
	electricity.US	1.67E+00	kWh/kg	[2]
	naturalgas_select	7.28E+00	MJ/kg	[2]
	tankertruck	4.26E-01	mt_km/kg	[2]
	rail	1.26E+00	mt_km/kg	[2]
	na_brine	5.83E+00	kg/kg	[2]
corn-stover				
	K2O	1.50E-02	kg/kg	[6]
	N	3.51E-03	kg/kg	[6]
	P2O5	2.51E-03	kg/kg	[6]
	diesel	2.60E-01	MJ/kg	[6]
	flatbedtruck	3.02E-02	mt_km/kg	[6]
na_brine				
	RFO	1.28E-01	MJ/kg	[2]
	electricity.US	2.56E-01	kWh/kg	[2]
	naturalgas_select	7.91E-01	MJ/kg	[2]
activated_carbon				
	coal	8.79E+01	MJ/kg	[15,16]
	electricity.US	1.84E+00	kWh/kg	[15,16]
	naturalgas_select	1.22E+01	MJ/kg	[15,16]
	flatbedtruck	7.00E-02	mt_km/kg	[15,16]
	barge	2.28E+01	mt_km/kg	[15,16]
KH2PO4				
	phosphoric-acid	1.04E+00	kg/kg	[6]
	koh	4.12E-01	kg/kg	[6]
KOH				
	diesel	1.41E+00	MJ/kg	[6]
	electricity.US	3.28E+00	kWh/kg	[6]
	naturalgas_select	1.22E+00	MJ/kg	[6]
	kcl	1.52E+00	kg/kg	[6]
KCl				
	RFO	1.41E+00	MJ/kg	[6]
	electricity.US	5.29E-01	kWh/kg	[6]
	naturalgas_select	1.22E+00	MJ/kg	[6]
NH4OH				
	ammonia_conventional	4.86E-01	kg/kg	[6]

	electricity.US	1.11E-03	kWh/kg	31
langbeinite				
	diesel	1.41E+00	MJ/kg	6
	electricity.US	5.29E-01	kWh/kg	6
	naturalgas_select	1.22E+00	MJ/kg	6
polypropylene				
	diesel	6.87E-02	MJ/kg	26
	rfo	8.10E-01	MJ/kg	26
	crudeoil	1.60E+01	MJ/kg	26
	electricity.US	3.88E-01	kWh/kg	26
	gasoline	3.50E-02	MJ/kg	26
	naturalgas_select	4.04E+01	MJ/kg	26
	flatbedtruck	3.09E-02	mt_km/kg	26
	gaspipeline	1.22E+00	mt_km/kg	26
	liquidpipeline	4.83E-01	mt_km/kg	26
	rail	2.41E-02	mt_km/kg	26
	barge	2.16E+00	mt_km/kg	26
	lpg	3.26E-04	kg/kg	26
	landfill_inorganics	4.17E-02	wet kg/kg	26

III. Eutrophication and Acidification Potential

For eutrophication potential, we only assess marine impacts and exclude freshwater impacts because marine ecosystems are nitrogen-limited and freshwater ecosystems are phosphorus-limited. We do not model or track any changes in phosphorous fluxes and therefore have insufficient information to expect or evaluate impacts on freshwater eutrophication from any of the biorefinery scenarios. The analysis of marine eutrophication potential only includes consideration of NH_3 and NO_X emissions to air because we assume that none of the biorefinery scenarios contribute to changes in nitrogen fluxes to water or soils. In all scenarios, we assume there is no leakage of nitrogenous compounds to water from the biorefinery. In codigestion scenarios, manure diversion from farms induces synthetic fertilizer use but we assume that the rate of nitrogen application remains the same. Because eutrophication characterization factors from the Tool for Reduction and Assessment of Chemicals and Other Environmental Impacts (TRACI v2.2) are spatially resolved, we evaluate eutrophication potential for 3 regional scenarios and use Iowa-specific, California-specific, and US-average factors (Table S7). Total eutrophication potential is reported in units of kg $N_{eq.}$.

Table S7. Marine Eutrophication Potential Characterization Factors from TRACI[32,33]

Pollutant	Characterization Factor			Units
	Iowa	California	US	
NH_3	7.31E-02	7.50E-02	1.00E-01	kg N eq. / kg N (NH3)
NO_X	9.42E-02	7.21E-02	9.30E-02	kg N eq. / kg N (NO2)

To estimate acidification potential, we consider impacts from gaseous emissions of NO_X, SO_2, NH_3 and H_2S. For NO_X, SO_2, and NH_3, we consider full life-cycle emissions. For H_2S, because of data availability, we only account for direct facility emissions (see Table S2). Relevant TRACI acidification potential characterization factors are listed in Table S8. Because TRACI does not provide an average characterization factor for NO_X, we use the characterization factor NO_2. Total acidification potential is reported in units of kg $SO_{2eq.}$.

Table S8. Acidification Potential Characterization Factors from TRACI[32,33]

Pollutant	Characterization Factor	Units
SO_2	1.00E+00	kg SO2 eq. / kg pollutant
NO_2	7.00E-01	kg SO2 eq. / kg pollutant
NH_3	1.88E+00	kg SO2 eq. / kg pollutant
H_2S	1.88E+00	kg SO2 eq. / kg pollutant

IV. Monte Carlo Sensitivity Analysis

We conduct a sensitivity analysis to explore potential variability in key emission sources throughout the study system using Monte Carlo simulations. In general, our standard approach is to apply a probability distribution function (PDF) to a specific emission factor using a triangular shape, a mode value that is equal to the standard model value (data in Table S5) and min/max values that are 20% less than or greater than the model value (see Table S9 and S10). We use this standard approach to explore potential variability of emissions related to electricity generation, waste diversion and direct combustion (Table S9 and S10). In the case of direct emissions from on-site biomass combustion, we vary non-GHG emission factors by 5% (instead of 20%) in a triangular PDF. In the case of methane leakage from the digester, we use literature values based on seasonal variability to vary methane leakage rate (Table S9).[34] Because emissions from conventional manure management (storage and field application) can vary so substantially, when possible, we incorporate specific emission factors from literature to use as min and/or max values in relevant PDFs (Table S9 and S10).[17,19,20] In other instances, we use a uniform PDF with a min value of zero and max value equal to the model value to avoid overestimating manure diversion benefits and maintaining a conservative approach to credits. This sensitivity analysis serves to better demonstrate the minimum benefits from manure diversion to a biorefinery rather than explore the maximum potential benefits from avoiding manure storage and field application.

Table S9. Monte Carlo Assumptions for Greenhouse Gas Emission Factors and Relevant Process Parameters

Units for all emission factors (min, mode, max) are kg of pollutant per unit indicated at the end of the unit process name. The mode is assumed to be the base value used in the original LCA model and the associated reference and source is provided in Table S5. The descriptions and sources provided in this table describe assumptions for the min and max values.

Pollutant	Unit Process with Emission Factor	Shape	Min	Mode (model value)	Max	Description	Source
ch4	swine_manure.storage.kg	triangular	1.60E-04	2.02E-04	1.53E-02	Min = -20%; max adapted from literature values	17
ch4	swine_manure.field_application.kg	triangular	9.97E-04	1.25E-03	2.45E-02	Min = -20%; max adapted from literature values	17
n2o	swine_manure.storage.kg	uniform	0.00E+00	-	1.69E-04	Conservative assumption: min = zero and max = model value	
n2o	swine_manure.field_application.kg	uniform	0.00E+00	-	1.50E-07	Conservative assumption: min = zero and max = model value	
ch4	dairy_manure.storage.kg	triangular	1.73E-03	4.05E-03	4.90E-03	Min and max adapted from literature values	19
ch4	dairy_manure.field_application.kg	uniform	1.30E-06	-	2.22E-05	Min and max adapted from literature values	19
n2o	dairy_manure.storage.kg	triangular	9.00E-06	2.02E-05	4.93E-05	+/- 20%	
n2o	dairy_manure.field_application.kg	triangular	3.04E-06	3.80E-06	5.70E-06	+/- 20%	
co2	electricity.US.kWh	triangular	3.31E-01	4.14E-01	4.97E-01	+/- 20%	
co2	electricity.MRO.kWh	triangular	3.30E-01	4.13E-01	4.96E-01	+/- 20%	
ch4	landfill_foodwaste.wet_kg	triangular	1.04E-02	1.30E-02	1.56E-02	+/- 20%	

co2	PLA.kg	triangular	6.62E-01	8.27E-01	9.92E-01	+/- 20%	
ch4	CNG_combust_heavydutytruck.MJ	triangular	1.44E-05	1.80E-05	2.16E-05	+/- 20%	
n2o	CNG_combust_heavydutytruck.MJ	triangular	4.00E-06	5.00E-06	6.00E-06	+/- 20%	
**Pollutant**	**Process Parameter**	**Shape**	**Min**	**Mode (model value)**	**Max**	**Description**	**Source**
ch4	digestion_leakage_rate.%	triangular	1.70%	3.10%	5.20%	Adapted from literature values; based on seasonal variability.	[34]

Table S10. Monte Carlo Assumptions for Air Pollutant Emission Factors

Units for all emission factors (min, mode, max) are kg of pollutant per unit indicated at the end of the unit process name. The mode is assumed to be the base value used in the original LCA model and the associated reference and source is provided in Table S5. The descriptions and sources provided in this table describe assumptions for the min and max values.

Pollutant	Unit Process (emission factor)	Shape	Min	Mode (model value)	Max	Description	Source
nh3	swine_manure.storage.kg	triangular	3.93E-05	2.37E-04	3.00E-03	Min and max adapted from literature values	[20]
nh3	swine_manure.field_application.kg	uniform	7.58E-05	-	4.36E-04	Min and max adapted from literature values	[20]
nh3	dairy_manure.field_application.kg	uniform	7.58E-05	-	5.09E-04	Min and max adapted from literature values	[20]
nh3	dairy_manure.storage.kg	triangular	3.93E-05	4.10E-05	2.09E-04	Min and max adapted from literature values	[20]
so2	electricity.US.kWh	triangular	1.98E-04	2.48E-04	2.98E-04	+/- 20%	
nox	electricity.US.kWh	triangular	1.91E-04	2.38E-04	2.86E-04	+/- 20%	
so2	electricity.MRO.kWh	triangular	2.58E-04	3.23E-04	3.88E-04	+/- 20%	
nox	electricity.MRO.kWh	triangular	2.53E-04	3.16E-04	3.79E-04	+/- 20%	
nox	corn.stover.kg	triangular	8.52E-05	1.07E-04	1.28E-04	+/- 20%	
pm25	corn.stover.kg	triangular	5.85E-06	7.31E-06	8.77E-06	+/- 20%	
voc	corn.stover.kg	triangular	7.70E-06	9.62E-06	1.15E-05	+/- 20%	
pm25	soybeanmeal.kg	triangular	2.80E-05	3.50E-05	4.20E-05	+/- 20%	
voc	soybeanmeal.kg	triangular	5.02E-04	6.28E-04	7.54E-04	+/- 20%	
nox	PLA.kg	triangular	9.84E-03	1.23E-02	1.23E-02	+/- 20%	
so2	PLA.kg	triangular	5.92E-03	7.40E-03	7.40E-03	+/- 20%	
voc	swine_manure.storage.kg	triangular	4.85E-05	6.06E-05	7.27E-05	+/- 20%	
voc	dairy_manure.storage.kg	triangular	4.85E-05	6.06E-05	7.27E-05	+/- 20%	
nox	CNG_combust_heavydutytruck.MJ	triangular	5.20E-05	6.50E-05	7.80E-05	+/- 20%	
pm25	biomass_combust.kg	triangular	4.36E-02	5.45E-02	6.54E-02	+/- 5%	
nox	biomass_combust.kg	triangular	5.25E-03	6.56E-03	7.87E-03	+/- 5%	
voc	biomass_combust.kg	triangular	1.27E-04	1.59E-04	1.91E-04	+/- 5%	

V. Tabulated LCA Results

Table S11. Life Cycle Greenhouse Gas Emissions

Units for all values are g $CO_{2eq.}$ per MJ ethanol produced.

Stage	Scenario 1: Baseline	Scenario 2: Codigestion + Electricity	Scenario 3A: Codigestion + RNG (fleet fueling)	Scenario 3B: Codigestion + RNG (SMR)	Scenario 4: Codigestion + SCP	Scenario 5A: Codigestion + PHB (offset PLA)	Scenario 5B: Codigestion + PHB (offset PP)
**Corn stover feedstock**	5.8	5.7	5.8	5.8	5.8	5.8	5.8
**Organic waste feedstock**	0.0	3.6	3.6	3.6	3.6	3.6	3.6
**Pretreatment**	35.9	31.2	37.0	37.0	40.1	40.1	40.1
**Fermentation**	1.5	1.3	1.5	1.5	1.7	1.7	1.7
**Ethanol recovery**	0.7	0.0	0.9	0.9	1.4	1.4	1.4
**Wastewater treatment**	7.7	12.6	15.2	15.2	16.6	17.8	17.8
**Onsite energy production**	0.5	0.5	0.7	0.7	1.0	1.0	1.0
**Utilities**	0.8	0.0	1.2	1.2	1.8	2.3	2.3
**Waste**	0.2	0.2	0.2	0.2	0.2	0.2	0.2
**Biogas upgrading**	0.0	0.0	1.4	1.4	0.0	0.0	0.0
**PHB production**	0.0	0.0	0.0	0.0	0.0	23.4	23.4
**SCP production**	0.0	0.0	0.0	0.0	21.6	0.0	0.0
**Food Waste Credit**	0.0	-9.4	-9.4	-9.4	-9.4	-9.4	-9.4
**Manure Credit**	0.0	-21.5	-21.5	-21.5	-21.5	-21.5	-21.5
**Electricity Credit**	0.0	-15.5	0.0	0.0	0.0	0.0	0.0
**Coproduct Credit**	0.0	0.0	-62.8	-46.1	-6.4	-6.4	-8.9
**Total**	**53.0**	**8.7**	**-26.1**	**-9.4**	**56.5**	**60.0**	**57.6**

Table S12. Life Cycle NH_3 Emissions

Units for all values are g NH_3 per MJ ethanol produced.

Stage	Scenario 1: Baseline	Scenario 2: Codigestion + Electricity	Scenario 3A: Codigestion + RNG (fleet fueling)	Scenario 3B: Codigestion + RNG (SMR)	Scenario 4: Codigestion + SCP	Scenario 5A: Codigestion + PHB (offset PLA)	Scenario 5B: Codigestion + PHB (offset PP)
**Corn stover feedstock**	-1.02E-21	-1.02E-21	-1.02E-21	-1.02E-21	-1.01E-21	-1.02E-21	-1.02E-21
**Organic waste feedstock**	0.00E+00	-3.09E-23	-3.09E-23	-3.09E-23	-3.09E-23	-3.09E-23	-3.09E-23
**Pretreatment**	-6.09E-22	-1.37E-22	-2.28E-22	-2.28E-22	-8.14E-22	-4.95E-22	-4.95E-22
**Fermentation**	-1.41E-22	-1.30E-22	-1.27E-22	-1.27E-22	-1.42E-22	-1.34E-22	-1.34E-22
**Ethanol recovery**	-5.67E-24	0.00E+00	-8.70E-24	-8.70E-24	2.20E-23	-2.05E-23	-2.05E-23
**Wastewater treatment**	1.61E-04	3.74E-04	3.74E-04	3.74E-04	3.74E-04	3.75E-04	3.75E-04
**Onsite energy production**	-3.36E-24	9.02E-25	-8.34E-24	-8.34E-24	-2.37E-23	-1.63E-23	-1.63E-23
**Utilities**	-1.02E-23	0.00E+00	2.67E-24	2.67E-24	5.33E-24	4.05E-25	4.05E-25
**Waste**	2.45E-23	-6.84E-24	-1.51E-22	-1.51E-22	-7.00E-23	-9.30E-23	-9.30E-23
**Biogas upgrading**	0.00E+00	0.00E+00	-5.02E-23	-5.02E-23	0.00E+00	0.00E+00	0.00E+00
**PHB production**	0.00E+00	0.00E+00	0.00E+00	0.00E+00	0.00E+00	3.50E-22	3.50E-22
**SCP production**	0.00E+00	0.00E+00	0.00E+00	0.00E+00	-5.35E-23	0.00E+00	0.00E+00
**Food Waste Credit**	0.00E+00	-3.63E-05	-3.63E-05	-3.63E-05	-3.63E-05	-3.63E-05	-3.63E-05
**Manure Credit**	0.00E+00	-5.66E-02	-5.66E-02	-5.66E-02	-5.66E-02	-5.66E-02	-5.66E-02
**Electricity Credit**	0.00E+00	-2.02E-22	0.00E+00	0.00E+00	0.00E+00	0.00E+00	0.00E+00
**Coproduct Credit**	0.00E+00	0.00E+00	5.59E-22	2.02E-24	0.00E+00	-1.95E-05	-7.70E-06
**Total**	**1.61E-04**	**-5.63E-02**	**-5.63E-02**	**-5.63E-02**	**-5.63E-02**	**-5.63E-02**	**-5.63E-02**

Table S13. Life Cycle VOC Emissions

Units for all values are g VOC per MJ ethanol produced.

Stage	Scenario 1: Baseline	Scenario 2: Codigestion + Electricity	Scenario 3A: Codigestion + RNG (fleet fueling)	Scenario 3B: Codigestion + RNG (SMR)	Scenario 4: Codigestion + SCP	Scenario 5A: Codigestion + PHB (offset PLA)	Scenario 5B: Codigestion + PHB (offset PP)
Corn stover feedstock	1.59E-03	1.59E-03	1.60E-03	1.60E-03	1.60E-03	1.60E-03	1.60E-03
Organic waste feedstock	0.00E+00	3.35E-04	3.35E-04	3.35E-04	3.35E-04	3.35E-04	3.35E-04
Pretreatment	1.68E-03	1.28E-03	1.77E-03	1.77E-03	2.04E-03	2.04E-03	2.04E-03
Fermentation	2.06E-04	1.88E-04	2.10E-04	2.10E-04	2.22E-04	2.22E-04	2.22E-04
Ethanol recovery	6.32E-05	0.00E+00	7.84E-05	7.84E-05	1.20E-04	1.21E-04	1.21E-04
Wastewater treatment	1.11E-04	5.92E-07	2.23E-04	2.23E-04	3.41E-04	4.41E-04	4.41E-04
Onsite energy production	4.11E-05	2.95E-05	5.77E-05	5.77E-05	7.84E-05	7.90E-05	7.90E-05
Utilities	6.53E-05	0.00E+00	1.04E-04	1.04E-04	1.50E-04	1.96E-04	1.96E-04
Waste	5.84E-06	8.64E-06	8.68E-06	8.68E-06	8.53E-06	8.55E-06	8.55E-06
Biogas upgrading	0.00E+00	0.00E+00	1.12E-04	1.12E-04	0.00E+00	0.00E+00	0.00E+00
PHB production	0.00E+00	0.00E+00	0.00E+00	0.00E+00	0.00E+00	2.20E-03	2.20E-03
SCP production	0.00E+00	0.00E+00	0.00E+00	0.00E+00	2.05E-03	0.00E+00	0.00E+00
Food Waste Credit	0.00E+00	-1.08E-06	-1.08E-06	-1.08E-06	-1.08E-06	-1.08E-06	-1.08E-06
Manure Credit	0.00E+00	-2.20E-02	-2.20E-02	-2.20E-02	-2.20E-02	-2.20E-02	-2.20E-02
Electricity Credit	0.00E+00	-1.31E-03	0.00E+00	0.00E+00	0.00E+00	0.00E+00	0.00E+00
Coproduct Credit	0.00E+00	0.00E+00	-4.98E-03	-4.33E-03	-8.06E-03	-2.13E-04	-3.03E-03
**Total**	**3.76E-03**	**-1.99E-02**	**-2.25E-02**	**-2.18E-02**	**-2.31E-02**	**-1.50E-02**	**-1.78E-02**

Table S14. Life Cycle NO_X Emissions

Units for all values are g NO_x per MJ ethanol produced.

Stage	Scenario 1: Baseline	Scenario 2: Codigestion + Electricity	Scenario 3A: Codigestion + RNG (fleet fueling)	Scenario 3B: Codigestion + RNG (SMR)	Scenario 4: Codigestion + SCP	Scenario 5A: Codigestion + PHB (offset PLA)	Scenario 5B: Codigestion + PHB (offset PP)
**Corn stover feedstock**	2.10E-02	2.10E-02	2.10E-02	2.10E-02	2.10E-02	2.10E-02	2.10E-02
**Organic waste feedstock**	0.00E+00	3.98E-03	3.98E-03	3.98E-03	3.98E-03	3.98E-03	3.98E-03
**Pretreatment**	1.34E-02	1.01E-02	1.43E-02	1.43E-02	1.65E-02	1.65E-02	1.65E-02
**Fermentation**	3.19E-03	3.05E-03	3.23E-03	3.23E-03	3.33E-03	3.33E-03	3.33E-03
**Ethanol recovery**	5.37E-04	0.00E+00	6.66E-04	6.66E-04	1.02E-03	1.02E-03	1.02E-03
**Wastewater treatment**	9.41E-04	4.76E-06	1.89E-03	1.89E-03	2.89E-03	3.75E-03	3.75E-03
**Onsite energy production**	1.14E-03	1.82E-03	1.76E-03	1.76E-03	1.95E-03	2.03E-03	2.03E-03
**Utilities**	5.55E-04	0.00E+00	8.83E-04	8.83E-04	1.27E-03	1.66E-03	1.66E-03
**Waste**	4.18E-05	6.18E-05	6.21E-05	6.21E-05	6.10E-05	6.12E-05	6.12E-05
**Biogas upgrading**	0.00E+00	0.00E+00	9.55E-04	9.55E-04	0.00E+00	0.00E+00	0.00E+00
**PHB production**	0.00E+00	0.00E+00	0.00E+00	0.00E+00	0.00E+00	2.00E-02	2.00E-02
**SCP production**	0.00E+00	0.00E+00	0.00E+00	0.00E+00	1.87E-02	0.00E+00	0.00E+00
**Food Waste Credit**	0.00E+00	-1.05E-05	-1.05E-05	-1.05E-05	-1.05E-05	-1.05E-05	-1.05E-05
**Manure Credit**	0.00E+00	1.63E-03	1.63E-03	1.63E-03	1.63E-03	1.63E-03	1.63E-03
**Electricity Credit**	0.00E+00	-1.11E-02	0.00E+00	0.00E+00	0.00E+00	0.00E+00	0.00E+00
**Coproduct Credit**	0.00E+00	0.00E+00	-2.83E-02	-1.40E-03	-6.19E-03	-6.08E-02	-5.24E-03
**Total**	**4.09E-02**	**3.05E-02**	**2.20E-02**	**4.89E-02**	**6.62E-02**	**1.43E-02**	**6.98E-02**

Table S15. Life Cycle SO_2 Emissions

Units for all values are g SO_2 per MJ ethanol produced.

Stage	Scenario 1: Baseline	Scenario 2: Codigestion + Electricity	Scenario 3A: Codigestion + RNG (fleet fueling)	Scenario 3B: Codigestion + RNG (SMR)	Scenario 4: Codigestion + SCP	Scenario 5A: Codigestion + PHB (offset PLA)	Scenario 5B: Codigestion + PHB (offset PP)
**Corn stover feedstock**	1.05E-03	1.01E-03	1.06E-03	1.06E-03	1.09E-03	1.09E-03	1.09E-03
**Organic waste feedstock**	0.00E+00	7.01E-04	7.01E-04	7.01E-04	7.01E-04	7.01E-04	7.01E-04
**Pretreatment**	1.21E-02	8.68E-03	1.29E-02	1.29E-02	1.51E-02	1.51E-02	1.51E-02
**Fermentation**	2.49E-03	2.34E-03	2.52E-03	2.52E-03	2.62E-03	2.62E-03	2.62E-03
**Ethanol recovery**	5.38E-04	0.00E+00	6.67E-04	6.67E-04	1.02E-03	1.03E-03	1.03E-03
**Wastewater treatment**	2.30E-02	1.00E-02	1.82E-02	1.82E-02	1.97E-02	2.12E-02	2.12E-02
**Onsite energy production**	2.31E-04	3.45E-05	3.08E-04	3.08E-04	4.74E-04	4.80E-04	4.80E-04
**Utilities**	5.56E-04	0.00E+00	8.86E-04	8.86E-04	1.27E-03	1.67E-03	1.67E-03
**Waste**	3.30E-05	4.88E-05	4.90E-05	4.90E-05	4.82E-05	4.83E-05	4.83E-05
**Biogas upgrading**	0.00E+00	0.00E+00	9.63E-04	9.63E-04	0.00E+00	0.00E+00	0.00E+00
**PHB production**	0.00E+00	0.00E+00	0.00E+00	0.00E+00	0.00E+00	2.70E-02	2.70E-02
**SCP production**	0.00E+00	0.00E+00	0.00E+00	0.00E+00	2.57E-02	0.00E+00	0.00E+00
**Food Waste Credit**	0.00E+00	-7.06E-04	-7.06E-04	-7.06E-04	-7.06E-04	-7.06E-04	-7.06E-04
**Manure Credit**	0.00E+00	1.62E-03	1.62E-03	1.62E-03	1.62E-03	1.62E-03	1.62E-03
**Electricity Credit**	0.00E+00	-1.11E-02	0.00E+00	0.00E+00	0.00E+00	0.00E+00	0.00E+00
**Coproduct Credit**	0.00E+00	0.00E+00	-1.23E-02	-6.41E-05	-3.76E-03	-3.66E-02	-8.78E-03
**Total**	**4.00E-02**	**1.26E-02**	**2.69E-02**	**3.91E-02**	**6.49E-02**	**3.53E-02**	**6.31E-02**

Table S16. Life Cycle $PM_{2.5}$ Emissions

Units for all values are g $PM_{2.5}$ per MJ ethanol produced.

Stage	Scenario 1: Baseline	Scenario 2: Codigestion + Electricity	Scenario 3A: Codigestion + RNG (fleet fueling)	Scenario 3B: Codigestion + RNG (SMR)	Scenario 4: Codigestion + SCP	Scenario 5A: Codigestion + PHB (offset PLA)	Scenario 5B: Codigestion + PHB (offset PP)
**Corn stover feedstock**	1.17E-03	1.17E-03	1.17E-03	1.17E-03	1.17E-03	1.17E-03	1.17E-03
**Organic waste feedstock**	0.00E+00	7.10E-05	7.10E-05	7.10E-05	7.10E-05	7.10E-05	7.10E-05
**Pretreatment**	1.07E-03	8.14E-04	1.13E-03	1.13E-03	1.30E-03	1.30E-03	1.30E-03
**Fermentation**	2.47E-04	2.36E-04	2.50E-04	2.50E-04	2.58E-04	2.58E-04	2.58E-04
**Ethanol recovery**	4.05E-05	0.00E+00	5.02E-05	5.02E-05	7.69E-05	7.72E-05	7.72E-05
**Wastewater treatment**	7.09E-05	3.38E-07	1.43E-04	1.43E-04	2.18E-04	2.82E-04	2.82E-04
**Onsite energy production**	2.50E-03	3.72E-03	3.84E-03	3.84E-03	3.89E-03	4.10E-03	4.10E-03
**Utilities**	4.18E-05	0.00E+00	6.66E-05	6.66E-05	9.58E-05	1.25E-04	1.25E-04
**Waste**	4.32E-06	6.39E-06	6.42E-06	6.42E-06	6.31E-06	6.32E-06	6.32E-06
**Biogas upgrading**	0.00E+00	0.00E+00	7.21E-05	7.21E-05	0.00E+00	0.00E+00	0.00E+00
**PHB production**	0.00E+00	0.00E+00	0.00E+00	0.00E+00	0.00E+00	2.04E-03	2.04E-03
**SCP production**	0.00E+00	0.00E+00	0.00E+00	0.00E+00	1.95E-03	0.00E+00	0.00E+00
**Food Waste Credit**	0.00E+00	-6.90E-05	-6.90E-05	-6.90E-05	-6.90E-05	-6.90E-05	-6.90E-05
**Manure Credit**	0.00E+00	1.62E-04	1.62E-04	1.62E-04	1.62E-04	1.62E-04	1.62E-04
**Electricity Credit**	0.00E+00	-8.37E-04	0.00E+00	0.00E+00	0.00E+00	0.00E+00	0.00E+00
**Coproduct Credit**	0.00E+00	0.00E+00	-9.74E-04	-4.60E-05	-4.53E-04	-9.59E-06	-4.38E-04
**Total**	**5.14E-03**	**5.27E-03**	**5.91E-03**	**6.84E-03**	**8.67E-03**	**9.52E-03**	**9.09E-03**

VI. Forecasting Life-Cycle GHG Emissions

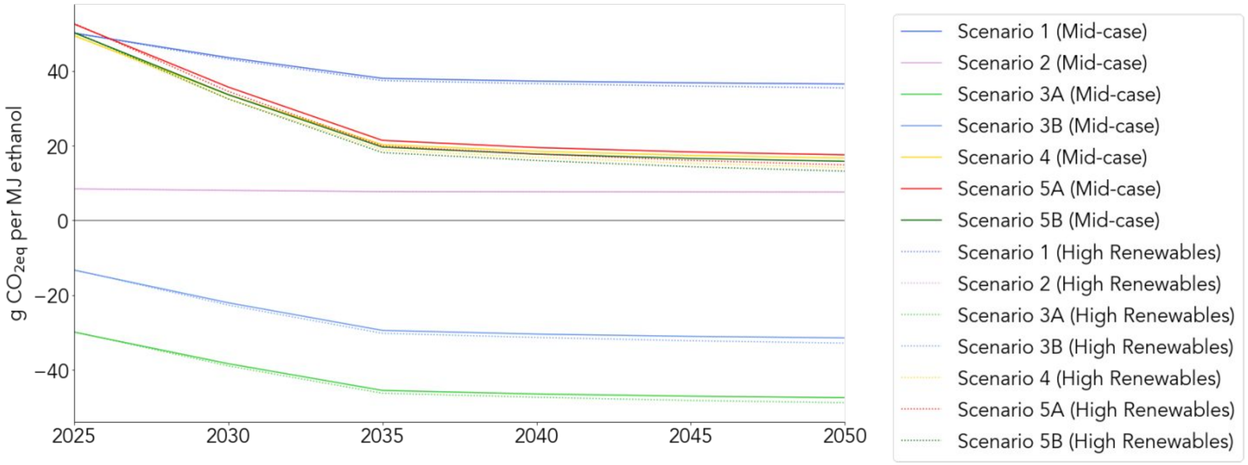

Figure S2. Greenhouse Gas Footprints Forecast Based on NREL's Cambium Datasets for the "Mid-case" and "High Renewables" US Electricity Sector Scenarios [35]

VII. Life-Cycle Marine Eutrophication and Acidification Potential

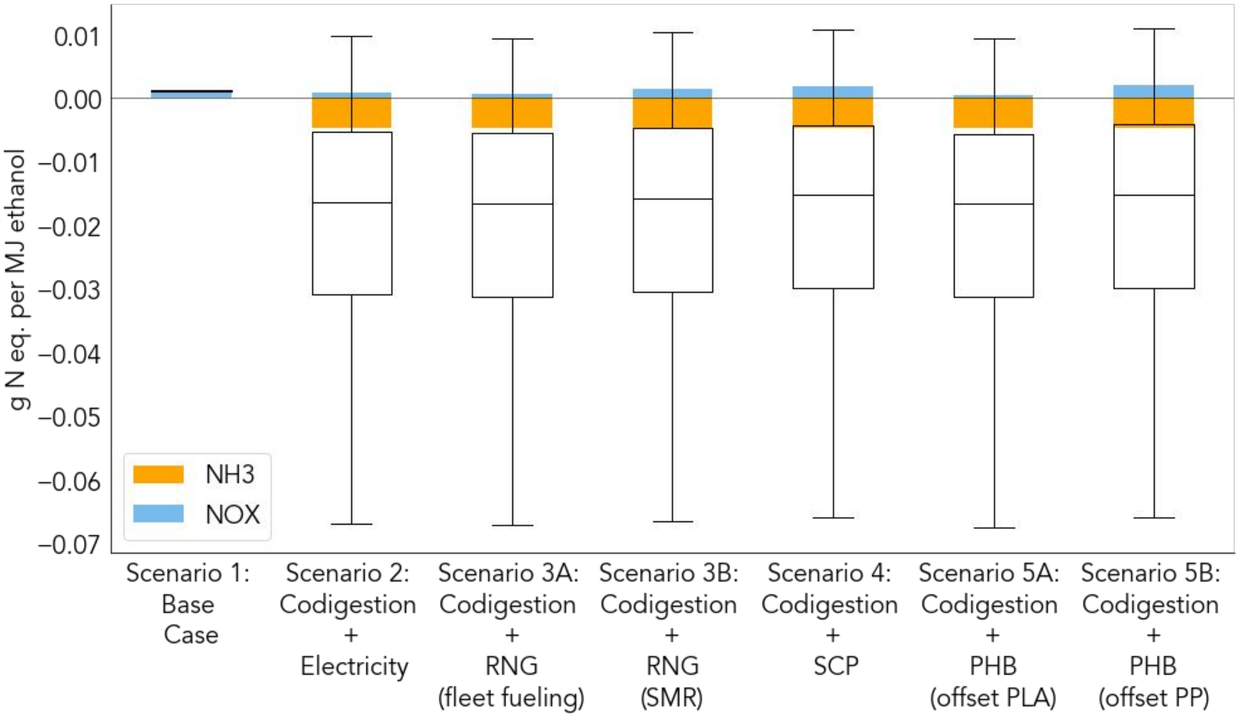

Figure S3. Net Effects of Each Scenario on System-Wide Life-Cycle Marine Eutrophication Potential Using US-Average Characterization Factors from TRACI2.2
The box and whisker plots demonstrate the variability of marine eutrophication potential based on the Monte Carlo simulations modeling life-cycle NH_3 and NO_X emissions (see Figure 2 in main text).

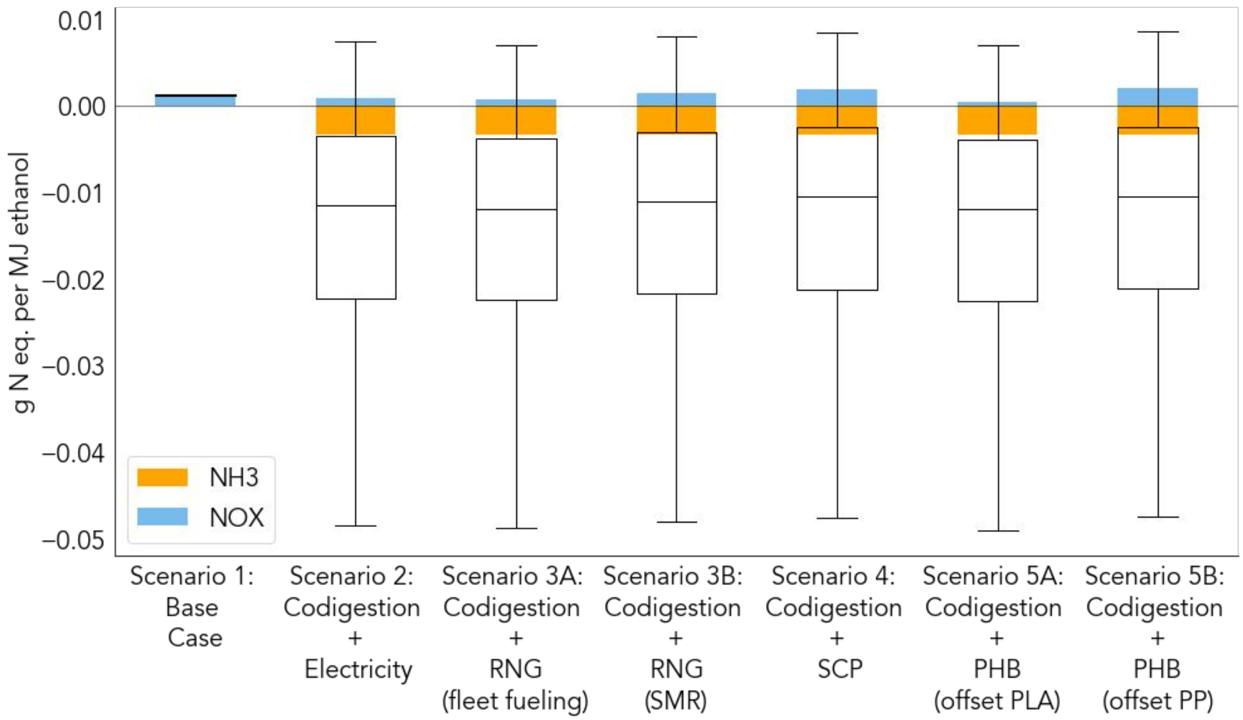

Figure S4. Net Effects of Each Scenario on System-Wide Life-Cycle Marine Eutrophication Potential Using Iowa-Specific Characterization Factors from TRACI2.2
The box and whisker plots demonstrate the variability of marine eutrophication potential based on the Monte Carlo simulations modeling life-cycle NH_3 and NO_X emissions (see Figure 2 in main text).

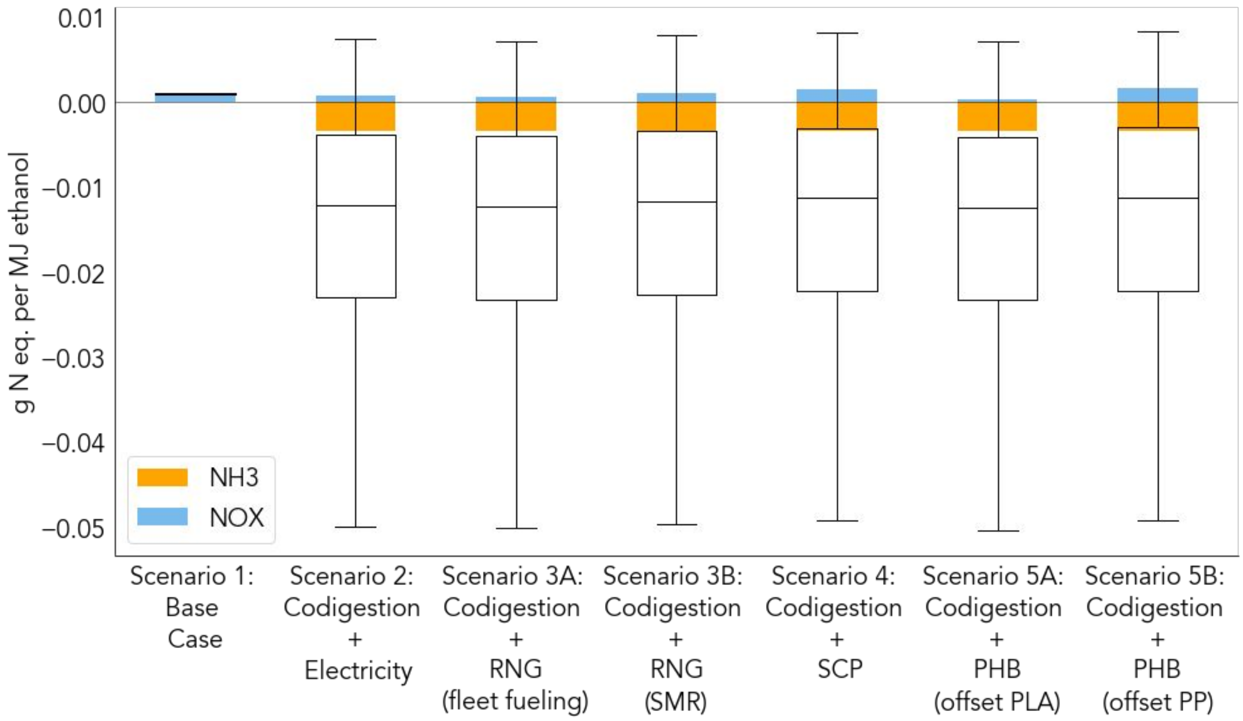

Figure S5. Net Effects of Each Scenario on System-Wide Life-Cycle Marine Eutrophication Potential Using California-Specific Characterization Factors from TRACI2.2

The box and whisker plots demonstrate the variability of marine eutrophication potential based on the Monte Carlo simulations modeling life-cycle NH_3 and NO_X emissions (see Figure 2 in main text).

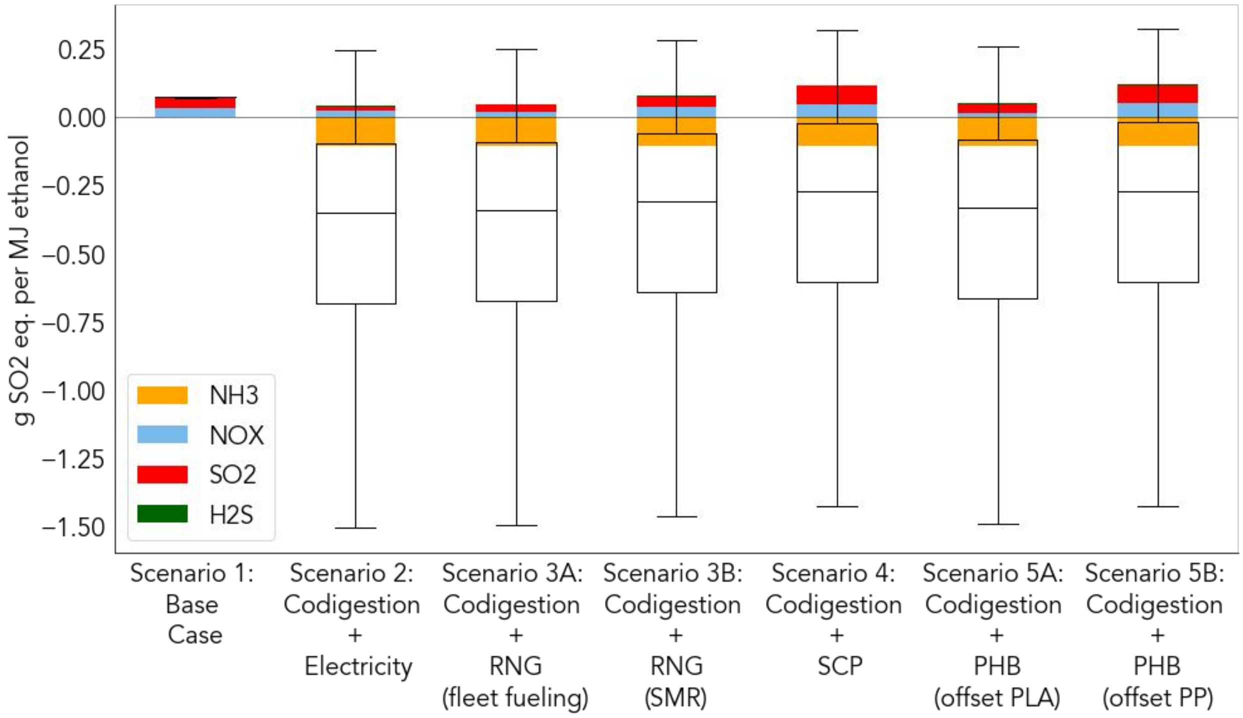

Figure S6. Net Effects of Each Scenario on System-Wide Life-Cycle Acidification Potential Using Characterization Factors from TRACI2.2

The box and whisker plots demonstrate the variability of marine eutrophication potential based on the Monte Carlo simulations modeling life-cycle NH_3 and NO_X emissions (see Figure 2 in main text).

VIII. Modeling human health impacts from air pollution using InMAP

The InMAP Source-Receptor Matrix (ISRM) was used to map changes in fine particulate matter ($PM_{2.5}$) concentrations and associated human health impacts[36]. This model considers how changes in primary $PM_{2.5}$ emissions as well as secondary formation from precursor emissions (i.e., NH_3, VOC, NO_X, and SO_2) affect local $PM_{2.5}$ concentrations. This occurs at a variable spatial resolution (between 1 and 48 km) depending on population. Within each grid cell is an associated population, allowing the model to link air pollution concentrations to health impacts through either the Krewski[37] or LePeule methods[38]. These methods consider how mortality rates change along with increases in air pollution concentrations. The output of this step was the expected mortality, which was translated to a monetary value through a value of a statistical life (VSL) assigned as $9 million. While ISRM allows for relative comparison between the health effects of different scenarios, there are limitations through using a reduced-form air quality model including older estimations of background concentrations and lower resolutions than traditional air quality models, particularly in rural areas due to the variable spatial resolution based on population.[36]

Table S17. Local Social Cost Analysis and Geographic Allocation Assumptions for Iowa

**Stages with local impacts**	**Emissions considered in local social cost analysis**	**Assumed emission location**	**Latitude**	**Longitude**	**Location description**
Corn Stover Sourcing	Combustion emissions from diesel use for corn stover collection.	Grace Hill, IA	41.263	-91.830	Arbitrary location in resource-rich area of biositing tool [39]
Feedstock Transportation	Direct emissions from the transportation of corn stover, organic waste feedstocks, and biorefinery waste.	Brighton, IA	41.172	-91.821	Small town close to Grace Hill
Direct Facility Emissions	Direct emissions from biogas and biomass combustion during onsite energy production. Fugitive emissions from wastewater treatment.	Brighton, IA	41.172	-91.821	Small town close to Grace Hill
Local Manure Diversion	Avoided emissions from manure storage and field application.	Grace Hill, IA	41.263	-91.830	Same assumptions as for corn stover sourcing[39]
Local Landfill Diversion	Avoided emissions from food waste landfilling.	Washington, IA	41.300	-91.693	Town close to facility area

Table S18. Geographic Allocation Assumptions for California

Stages with local impacts	Assumed emission locations	Latitude	Longitude	Location description
Corn Stover Sourcing	Lindsay	36.242	-119.198	Arbitrary location in resource (agricultural waste and manure)-rich area of biositing tool [39]
Feedstock Transportation	Kingsburg	36.460	-119.527	Town close to corn stover and manure locations
Direct Facility Emissions	Kingsburg	36.460	-119.527	Town close to corn stover and manure locations
Local Manure Diversion	Delano	35.899	-119.213	Arbitrary location in resource (agricultural waste and manure)-rich area of biositing tool [39]
Local Landfill Diversion	Resource Management Agency-Visalia Landfill	36.386	-119.379	Landfill in facility area

Table S19. Emissions Considered in Local Social Cost Analysis

These emissions, along with geographic coordinates from Tables S15 and S16, are used as ISRM inputs.

Scenario	Stage	g of pollutant emitted locally per MJ ethanol produced				
		NO_X	SO_2	VOC	NH_3	$PM_{2.5}$
**Scenario 1: Baseline**	Corn Stover Sourcing	1.37E-02	1.44E-05	1.24E-03	0.00E+00	9.41E-04
	Feedstock and Waste Transportation	1.16E-04	1.29E-05	0.00E+00	0.00E+00	1.29E-05
	Direct Facility Emissions	3.01E-04	2.20E-02	8.08E-06	1.61E-04	2.47E-03
	**Total Local Air Pollution**	**1.41E-02**	**2.21E-02**	**1.25E-03**	**1.61E-04**	**3.43E-03**
**Scenario 2: Codig + Elec**	Corn Stover Sourcing	1.37E-02	1.44E-05	1.24E-03	0.00E+00	9.41E-04
	Feedstock Transportation	3.69E-03	3.30E-05	2.04E-04	0.00E+00	4.64E-05
	Direct Facility Emissions	4.51E-04	1.01E-02	1.31E-05	3.74E-04	3.71E-03
	Local Manure Diversion	0.00E+00	0.00E+00	-2.22E-02	-5.66E-02	0.00E+00
	Local Landfill Diversion	-1.05E-05	-7.06E-04	-1.08E-06	-3.63E-05	-6.90E-05
	**Total Local Air Pollution**	**1.78E-02**	**9.39E-03**	**-2.08E-02**	**-5.63E-02**	**4.63E-03**
**Scenario 3A: Codig + CNG**	Corn Stover Sourcing	1.37E-02	1.44E-05	1.24E-03	0.00E+00	9.41E-04
	Feedstock Transportation	-2.09E-02	2.87E-05	-2.61E-03	0.00E+00	-2.81E-04
	Direct Facility Emissions	4.61E-04	1.64E-02	1.21E-05	3.74E-04	3.81E-03
	Local Manure Diversion	0.00E+00	0.00E+00	-2.22E-02	-5.66E-02	0.00E+00
	Local Landfill Diversion	-1.05E-05	-7.06E-04	-1.08E-06	-3.63E-05	-6.90E-05
	**Total Local Air Pollution**	**-6.76E-03**	**1.57E-02**	**-2.36E-02**	**-5.63E-02**	**4.40E-03**
**Scenario 3B: Codig + SMR**	Corn Stover Sourcing	1.37E-02	1.44E-05	1.24E-03	0.00E+00	9.41E-04
	Feedstock Transportation	3.69E-03	3.30E-05	2.04E-04	0.00E+00	4.65E-05
	Direct Facility Emissions	4.61E-04	1.64E-02	1.21E-05	3.74E-04	3.81E-03
	Local Manure Diversion	0.00E+00	0.00E+00	-2.22E-02	-5.66E-02	0.00E+00
	Local Landfill Diversion	-1.05E-05	-7.06E-04	-1.08E-06	-3.63E-05	-6.90E-05
	**Total Local Air Pollution**	**1.79E-02**	**1.57E-02**	**-2.08E-02**	**-5.63E-02**	**4.72E-03**
**Scenario 4: Codig + SCP**	Corn Stover Sourcing	1.37E-02	1.44E-05	1.24E-03	0.00E+00	9.41E-04
	Feedstock Transportation	3.69E-03	3.30E-05	2.04E-04	0.00E+00	4.64E-05
	Direct Facility Emissions	4.67E-04	1.68E-02	1.30E-05	3.74E-04	3.84E-03
	Local Manure Diversion	0.00E+00	0.00E+00	-2.22E-02	-5.66E-02	0.00E+00
	Local Landfill Diversion	-1.05E-05	-7.06E-04	-1.08E-06	-3.63E-05	-6.90E-05
	**Total Local Air Pollution**	**1.79E-02**	**1.62E-02**	**-2.08E-02**	**-5.63E-02**	**4.76E-03**
**Scenario 5: Codig + PHB (applies to both subscenarios A and B)**	Corn Stover Sourcing	1.37E-02	1.44E-05	1.24E-03	0.00E+00	9.41E-04
	Feedstock Transportation	3.69E-03	3.30E-05	2.04E-04	0.00E+00	4.64E-05
	Direct Facility Emissions	4.90E-04	1.75E-02	1.28E-05	3.75E-04	4.06E-03
	Local Manure Diversion	0.00E+00	0.00E+00	-2.22E-02	-5.66E-02	0.00E+00
	Local Landfill Diversion	-1.05E-05	-7.06E-04	-1.08E-06	-3.63E-05	-6.90E-05
	**Total Local Air Pollution**	**1.79E-02**	**1.68E-02**	**-2.08E-02**	**-5.63E-02**	**4.98E-03**

Table S20. ISRM Results

Location	Scenario #	Local Human Health Cost Per 1000 MJ Ethanol Produced				Local Human Health Cost Per Gallon Ethanol Produced			
		Total Deaths (Krewski)	Total Deaths (LePeule)	Damages (Krewski)	Damages (LePeule)	Total Deaths (Krewski)	Total Deaths (LePeule)	Damages (Krewski)	Damages (LePeule)
Iowa	1	1.07E-07	2.40E-07	$0.96	$2.16	9.46E-09	2.13E-08	$0.09	$0.19
	2	-1.76E-07	-3.95E-07	-$1.58	-$3.56	-1.56E-08	-3.51E-08	-$0.14	-$0.32
	3A	-2.01E-07	-4.53E-07	-$1.81	-$4.08	-1.79E-08	-4.02E-08	-$0.16	-$0.36
	3B	-1.57E-07	-3.53E-07	-$1.41	-$3.18	-1.39E-08	-3.13E-08	-$0.13	-$0.28
	4	-1.55E-07	-3.50E-07	-$1.40	-$3.15	-1.38E-08	-3.10E-08	-$0.12	-$0.28
	5	-1.53E-07	-3.43E-07	-$1.37	-$3.09	-1.35E-08	-3.04E-08	-$0.12	-$0.27
California	1	1.09E-07	2.46E-07	$0.98	$2.21	9.69E-09	2.18E-08	$0.09	$0.20
	2	-1.48E-07	-3.33E-07	-$1.33	-$3.00	-1.31E-08	-2.95E-08	-$0.12	-$0.27
	3A	-1.75E-07	-3.95E-07	-$1.58	-$3.55	-1.56E-08	-3.50E-08	-$0.14	-$0.32
	3B	-1.33E-07	-2.99E-07	-$1.20	-$2.70	-1.18E-08	-2.66E-08	-$0.11	-$0.24
	4	-1.32E-07	-2.96E-07	-$1.19	-$2.67	-1.17E-08	-2.63E-08	-$0.11	-$0.24
	5	-1.28E-07	-2.88E-07	-$1.15	-$2.59	-1.14E-08	-2.56E-08	-$0.10	-$0.23

Results presented in the main text (Figure 4) were determined using the LePeule method and assuming the biorefinery site to be in rural Iowa (IA). To show variability in social cost results from ISRM, we present additional ISRM results in the figure below considering the Krewski method in addition to the LePeule method and considering an example California (CA) location in addition to the Iowa location (Fig S6).

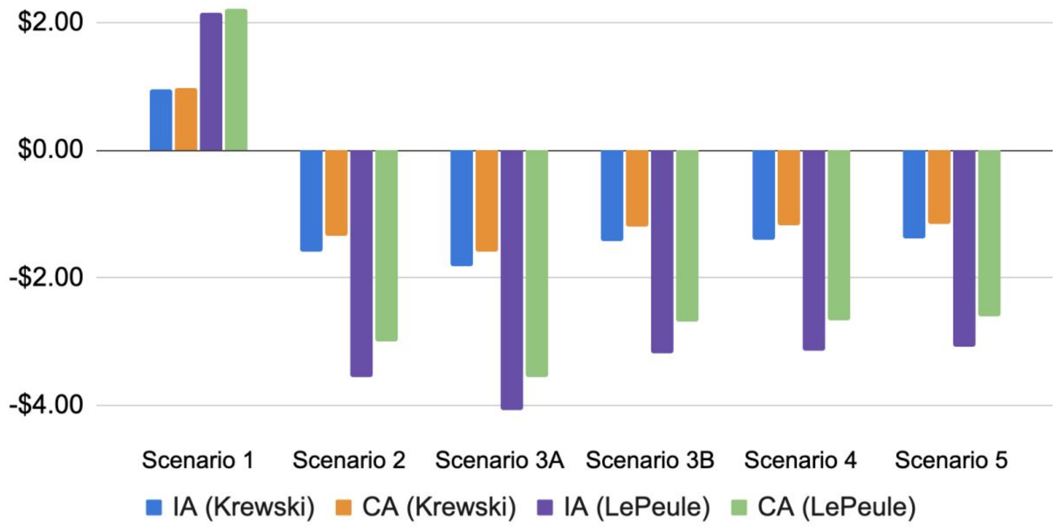

Figure S7. Human health damages from local air pollution per 1000 MJ ethanol produced for Iowa and California

References

(1) Wang, Y.; Baral, N. R.; Yang, M.; Scown, C. D. Co-Processing Agricultural Residues and Wet Organic Waste Can Produce Lower-Cost Carbon-Negative Fuels and Bioplastics. *Environ. Sci. Technol.* **2023**, *57*, 2958–2969.

(2) Wang, M.; Elgowainy, A.; Lee, U.; Bafana, A.; Benavides, P.; Burnham, A.; Cai, H.; Dai, Q.; Gracida, U.; Hawkins, T.; et al. Greenhouse gases, Regulated Emissions, and Energy use in Technologies Model ® (2020 Excel). *Argonne National Laboratory (ANL), Argonne, IL (United States)* **2020**.

(3) Wang, M.; Elgowainy, A.; Lee, U.; Benavides, P.; Burnham, A.; Cai, H.; Dai, Q.; Hawkins, T.; Kelly, J.; Kwon, H.; et al. Greenhouse gases, Regulated Emissions, and Energy use in Transportation Model ® (2018 Excel). *Argonne National Laboratory (ANL), Argonne, IL (United States)* **2018**.

(4) Jubb, C.; Nakhutin, A.; Cianci, V. C. S. Section 3.2: Ammonia Production, Chapter 3: Chemical Industry Emissions. In *2006 IPCC Guidelines for National Greenhouse Gas Inventories*; IPCC, 2006; Vol. 3: Industrial Processes and Product Use.

(5) Wang, M.; Elgowainy, A.; Lee, U.; Benavides, P.; Burnham, A.; Cai, H.; Dai, Q.; Hawkins, T.; Kelly, J.; Kwon, H.; et al. Greenhouse gases, Regulated Emissions, and Energy use in Transportation Model ® (2019 Excel). *Argonne National Laboratory (ANL), Argonne, IL (United States)* **2019**.

(6) Wang, M.; Elgowainy, A.; Lee, U.; Baek, K.; Bafana, A.; Benavides, P.; Burnham, A.; Cai, H.; Cappello, V.; Chen, P.; et al. Greenhouse gases, Regulated Emissions, and Energy use in Technologies Model ® (2022 Excel). *Argonne National Laboratory (ANL), Argonne, IL (United States)* **2022**.

(7) EPA. *Emissions & Generation Resource Integrated Database (eGRID)*; U.S.

Environmental Protection Agency, 2022.
(8) Spath, P.; Mann, M. *Life Cycle Assessment of Hydrogen Production via Natural Gas Steam Reforming*; NREL/TP-570-27637; National Renewable Energy Laboratory, 2001.
(9) Cohon, J. L.; Cropper, M. L.; Cullen, M. R.; English, M. R.; Field, C. B.; Greenbaum, D. S.; Hammitt, J. K.; Henderson, R. F.; Kling, C. L.; Krupnick, A. J.; et al. *Hidden costs of energy: unpriced consequences of energy production and use*; National Academies Press: Washington, D.C., 2010.
(10) EPA. *Inventory of U.S. Greenhouse Gas Emissions and Sinks: 1990 - 2011*; 430-R-13001; U.S. Environmental Protection Agency, 2013.
(11) Wang, M.; Elgowainy, A.; Lee, U.; Bafana, A.; Banerjee, S.; Benavides, P.; Bobba, P.; Burnham, A.; Cai, H.; Gracida, U.; et al. Greenhouse gases, Regulated Emissions, and Energy use in Technologies Model ® (2021 Excel). *Argonne National Laboratory (ANL), Argonne, IL (United States)* **2021**.
(12) Behera, S. K.; Park, J. M.; Kim, K. H.; Park, H.-S. Methane production from food waste leachate in laboratory-scale simulated landfill. *Waste Manag.* **2010**, *30*, 1502–1508.
(13) Alexander, A.; Burklin, C. E.; Singleton, A. *Landfill gas emissions model (LandGEM) version 3.02 user's guide*; Office of Research and Development, U.S. Environmental Protection Agency, 2005.
(14) Stokes, J.; Horvath, A. Life-Cycle Assessment of Urban Water Provision: Tool and Case Study in California. *J. Infrastruct. Syst.* **2011**, *17*, 15–24.
(15) Wernet, G.; Bauer, C.; Steubing, B.; Reinhard, J.; Moreno-Ruiz, E.; Weidema, B. The ecoinvent database version 3 (part I): overview and methodology. *Int. J. Life Cycle Assess.* **2016**, *21*, 1218–1230.
(16) Vilén, A.; Laurell, P.; Vahala, R. Comparative life cycle assessment of activated carbon production from various raw materials. *J. Environ. Manage.* **2022**, *324*, 116356.
(17) Wang, Y.; Dong, H.; Zhu, Z.; Gerber, P. J.; Xin, H.; Smith, P.; Opio, C.; Steinfeld, H.; Chadwick, D. Mitigating Greenhouse Gas and Ammonia Emissions from Swine Manure Management: A System Analysis. *Environ. Sci. Technol.* **2017**, *51*, 4503–4511.
(18) Nordahl, S. L.; Preble, C. V.; Kirchstetter, T. W.; Scown, C. D. Greenhouse Gas and Air Pollutant Emissions from Composting. *Environ. Sci. Technol.* **2023**, *57*, 2235–2247.
(19) Amon, B.; Kryvoruchko, V.; Amon, T.; Zechmeister-Boltenstern, S. Methane, nitrous oxide and ammonia emissions during storage and after application of dairy cattle slurry and influence of slurry treatment. *Agric. Ecosyst. Environ* **2006**, *112*, 153–162.
(20) Wu, D.; Zhang, Y.; Dong, G.; Du, Z.; Wu, W.; Chadwick, D.; Bol, R. The importance of ammonia volatilization in estimating the efficacy of nitrification inhibitors to reduce N2O emissions: A global meta-analysis. *Environ. Pollut.* **2021**, *271*, 116365.
(21) Vink, E. T. H.; Davies, S.; Kolstad, J. J. ORIGINAL RESEARCH: The eco-profile for current Ingeo® polylactide production. *Industrial Biotechnology* **2010**, *6*, 212–224.
(22) Adviento-Borbe, M. A. A.; Kaye, J. P.; Bruns, M. A.; McDaniel, M. D.; McCoy, M.; Harkcom, S. Soil greenhouse gas and ammonia emissions in long-term maize-based cropping systems. *Soil Sci. Soc. Am. J.* **2010**, *74*, 1623–1634.
(23) Hult, C.; Winnes, H. *Emission factors for methane engines on vehicles and ships*; Swedish Environmental Emissions Data; Swedish Environmental Protection Agency, 2020.

(24) Preble, C. V.; Chen, S. S.; Hotchi, T.; Sohn, M. D.; Maddalena, R. L.; Russell, M. L.; Brown, N. J.; Scown, C. D.; Kirchstetter, T. W. Air pollutant emission rates for dry anaerobic digestion and composting of organic municipal solid waste. *Environ. Sci. Technol.* **2020**, *54*, 16097–16107.
(25) Bhatt, A.; Ravi, V.; Zhang, Y.; Heath, G.; Davis, R.; Tan, E. C. D. Emission factors of industrial boilers burning biomass-derived fuels. *J. Air Waste Manag. Assoc.* **2023**, *73*, 241–257.
(26) Franklin Associates. *Cradle-to-grave life cycle inventory of nine plastic resins and four polyurethane precursors*; Plastics Division of the American Chemistry Council, 2011.
(27) Wang, M.; Elgowainy, A.; Lee, U.; Baek, K.; Balchandani, S.; Benavides, P.; Burnham, A.; Cai, H.; Chen, P.; Gan, Y.; et al. Greenhouse gases, Regulated Emissions, and Energy use in Technologies Model ® (2023 Excel). *Argonne National Laboratory (ANL), Argonne, IL (United States)* **2023**.
(28) Taylor, P.; Lavagne d'Ortigue, O.; Trudeau, N.; Francoeur, M. *Energy Efficiency Indicators for Public Electricity Production from Fossil Fuels*; International Energy Agency, 2008.
(29) Lu, Z.; Han, J.; Wang, M.; Cai, H.; Sun, P.; Dieffenthaler, D.; Gordillo, V.; Monfort, J.-C.; He, X.; Przesmitzki, S. Well-to-Wheels Analysis of the Greenhouse Gas Emissions and Energy Use of Vehicles with Gasoline Compression Ignition Engines on Low Octane Gasoline-Like Fuel. *SAE Int. J. Fuels Lubr.* **2016**, *9*, 527–545.
(30) Scown, C. D.; Nazaroff, W. W.; Mishra, U.; Strogen, B.; Lobscheid, A. B.; Masanet, E.; Santero, N. J.; Horvath, A.; McKone, T. E. Lifecycle greenhouse gas implications of US national scenarios for cellulosic ethanol production. *Environmental Research Letters* **2012**, *7*, 014011.
(31) Kim, S.; Overcash, M. Energy in chemical manufacturing processes: gate-to-gate information for life cycle assessment. *J. Chem. Technol. Biotechnol.* **2003**, *78*, 995–1005.
(32) Bare, J. TRACI 2.0: the tool for the reduction and assessment of chemical and other environmental impacts 2.0. *Clean Techn. Environ. Policy* **2011**, *13*, 687–696.
(33) Henderson, A. D.; Niblick, B.; Golden, H. E.; Bare, J. C. Modeling spatially resolved characterization factors for eutrophication potential in life cycle assessment. *Int. J. Life Cycle Assess.* **2021**, *26*, 1832–1846.
(34) Flesch, T. K.; Desjardins, R. L.; Worth, D. Fugitive methane emissions from an agricultural biodigester. *Biomass and Bioenergy* **2011**, *35*, 3927–3935.
(35) Gagnon, P.; Perez, P. A. S.; Florez, J.; Morris, J.; Velasquez, M. L.; Eisenman, J. *Cambium 2024 Data*; National Renewable Energy Laboratory, 2024.
(36) Tessum, C. W.; Apte, J. S.; Goodkind, A. L.; Muller, N. Z.; Mullins, K. A.; Paolella, D. A.; Polasky, S.; Springer, N. P.; Thakrar, S. K.; Marshall, J. D.; et al. Inequity in consumption of goods and services adds to racial-ethnic disparities in air pollution exposure. *Proc Natl Acad Sci USA* **2019**, *116*, 6001–6006.
(37) Krewski, D.; Jerrett, M.; Burnett, R. T.; Ma, R.; Hughes, E.; Shi, Y.; Turner, M. C.; Pope, C. A.; Thurston, G.; Calle, E. E.; et al. Extended follow-up and spatial analysis of the American Cancer Society study linking particulate air pollution and mortality. *Res Rep Health Eff Inst* **2009**, 5–114; discussion 115.

(38) Lepeule, J.; Laden, F.; Dockery, D.; Schwartz, J. Chronic exposure to fine particles and mortality: an extended follow-up of the Harvard Six Cities study from 1974 to 2009. *Environ. Health Perspect.* **2012**, *120*, 965–970.
(39) Huntington, T.; Baral, N.; Moore, M.; Nordahl, S.; Hendrickson, T.; Breunig, H.; Kavvada, O.; Wang, M.; Cui, X.; Scown, C.; et al. Biositing Webtool, v2. *Lawrence Berkeley National Laboratory (LBNL), Berkeley, CA (United States)* **2024**.
